# Supplementary material for: Quantifying spectral information about source separation in multisource odour plumes
Source: PLoS One. 2025 Jan 10;20(1):e0297754. doi: 10.1371/journal.pone.0297754 (PMC11723556; doi:10.1371/journal.pone.0297754)
Supplement: S1 File — (PDF) [file pone.0297754.s001.pdf]

Supporting Information

S1 Using other windows

To show that our results hold for other leakage-reducing windows, in Fig S7 and Fig S8 below we plot two panels from the Main Text but computed for a Kaiser window with parameter set to 16.

S2 Governing equations and parameterization of plumes

The 2D turbulent non-dimensional flow field $\mathbf{u}^* = [u^*, v^*]$ with streamwise (x^*) and cross-stream (y^*) velocity components u^* and v^* is governed by the non-dimensional, incompressible, Navier-Stokes and continuity equations

$$\frac{\partial \mathbf{u}^*}{\partial t^*} + \mathbf{u}^* \cdot \nabla^* \mathbf{u}^* = -\nabla^* p^* + \mathrm{Re}^{-1} \nabla^{*2} \mathbf{u}^* + \mathbf{g}^* \tag{S1}$$

{navier-stok

and

$$\nabla^* \cdot \mathbf{u}^* = 0 \; , \tag{S2}$$

{continuity}

where length, time, velocity, and pressure have been non-dimensionalized by ϕ, ϕ/U_o, U_o, and ρU_o^2, respectively. U_o is the mean ambient flow speed, ϕ is the characteristic length scale of the mixing grid (introduced below), and $\mathbf{g}^* = \phi \mathbf{g}/U^2$. Here and elsewhere we denote vector quantities in bold face, scalar quantities in plain face, and non-dimensional quantities with an asterisk. Note the Reynolds number Re describes the relative importance of inertial and viscous flow effects and parameterizes the flow field governed by Eqns. S1 and S2,

$$\mathrm{Re} = \frac{UL}{\nu} \; , \tag{S3}$$

{reynoldsNur

based on a characteristic velocity U (mean ambient flow speed U_o here), length scale (L), and the fluid kinematic viscosity ν. We take the mixing grid length scale ϕ (*pitch*, same order of magnitude as both cylinder diameters) as an appropriate characteristic length scale. The non-dimensional passive scalar concentration fields c^* is governed by the coupled non-dimensional, non-reacting advection-diffusion equation

$$\frac{\partial c^*}{\partial t^*} + \mathbf{u}^* \cdot \nabla^* c^* = \mathrm{Pe}^{-1} \nabla^{*2} c^* ; \tag{S4}$$

{advection-c

where scalar concentration c has been normalized by the source concentration c_o. Velocity and time are non-dimensionalized as in Eqns. S1 and S2. The Péclet number Pe parameterizes the scalar concentration field governed by Eq. S4, representing the relative importance of advective and diffusive odour mass transport as

$$\mathrm{Pe} = \frac{UL}{D} = \mathrm{ReSc} \; , \tag{S5}$$

{pecletNumbe

where the Schmidt number Sc is the ratio of the momentum diffusivity of the ambient fluid ν (i.e. kinematic viscosity) to the mass diffusivity of the given odour species in the ambient fluid D. Note that the instantaneous fluid velocities $\mathbf{u}^*$ and odour concentrations c^* are decomposed into mean ($\overline{\mathbf{u}^*}$, $\overline{c^*}$) and fluctuating (turbulent) components ($\mathbf{u}^{*'}$, $c^{*'}$) for analysis (the Reynolds decomposition).

Relevant properties of the flow field for our primary CFD simulation are plotted in Fig S1. As shown in the first column of panel A, the time-averaged velocity ($\overline{u}$, $\overline{v}$) is dominated by positive x-direction flow on the same order as the uniform inlet flow, with relatively small y-direction components. Clear boundary effects are visible near the top and bottom walls, which are observed in all plots in the figure. Such boundary effects are expected given the no-slip boundary conditions (i.e. top and bottom boundaries modeled as solid walls with 0 flow velocity at the boundary), which informed our selection of interior probe locations for the Fisher Information analysis that are well within the core region of the flow simulation.

The second column of panel A shows the integral length scales of the flow, computed using the approach detailed in Methods Sec 4.2.8. Physically, integral scales generally represent the size of the largest eddies

present in the flow. Note that for these plots, u is correlated in the streamwise (x) direction and v is correlated in the cross-stream (y) direction, as correlations were very small for u cross-stream and for v streamwise. The plots demonstrate an increase in length scales from the domain inlet to the outlet, as smaller-scale eddies coalesce into larger-scale structures downstream.

Panel B shows two quantities related to turbulence characteristics of the flow, turbulent isotropy and turbulent kinetic energy, derived from the Reynolds decomposition of the flow field into its time-average $(\overline{u_i})$ and fluctuating components $(u_i' = u_i - \overline{u_i})$. Turbulent kinetic energy was computed as:

$$k = \frac{1}{2}\left(\overline{u'^2} + \overline{v'^2}\right).$$

The bottom plot of panel B shows a high amount of energy generated by the cylinder array, which decays as the flow travels downstream. We computed the Reynolds stress tensor to investigate the isotropy of the flow, using the following definition of Reynolds stress components for constant-density flows:

$$\tau_{ij}'' \equiv \overline{u_i' u_j'}$$

The aspect ratio of the normal components of the Reynolds stress ellipse was used to produce a convenient visualization of the turbulence isotropy. The aspect ratio at each point was computed as $A = \min(\tau_{ij}'')/\max(\tau_{ij}'')$, with a maximum value of unity corresponding to a perfectly circular ellipse, which represents isotropic turbulence. As shown in the top plot of panel B, the flow field consists of interspersed isotropic zones $(A = 1)$ and mildly anisotropic zones $(0.5 < A < 1)$, with the exception of the highly anisotropic zones near the walls due to the aforementioned boundary effects.

S3 Additional simulations

S3.1 A second set of CFD simulations

Here we provide the details the second set of computational fluid dynamics simulations that we ran to further test our approach.

The domain schematic is shown in Fig S9. We simulated the flow of a fluid inside a two-dimensional wind-tunnel 1.2 m long and 1 m, uniformly meshed with cells of diameter ∼2 mm. The fluid parameters were the same as those of the simulations in the Main Text. Specifically, the fluid flowed at a velocity of 0.1 m/s, with kinematic viscosity of 1.8×10^{-5} m^2/s.

Vorticity was introduced into the fluid by twelve cylindrical obstacles of 38 mm diameter, equally spaced vertically with a gap of 38 mm at a horizontal distance of 20 cm from the inlet. Taking the gap between obstacles as the length scale yielded a Reynolds number of ∼200.

Point sources of odour (actual diameters were that of the cells of the mesh, ∼2 mm) were placed downstream of the obstacles at a horizontal distance of 40 cm from the inlet at various vertical locations. Just like the simulations in the Main Text, we simulated 16 odour sources, located symmetrically around the midline and spaced 8 mm apart, similar to the 7.5 mm spacing used in the Main Text. Like those simulations, the molecular diffusivity of odours was 1.5×10^{-5} m^2/s. Fluid flow over these odours sources carried odour downstream where their concentration profiles were detected by simulated probes.

The simulations in the Main Text were carried out using COMSOL. The supplementary simulations were performed in OpenFOAM [1], using the PISO algorithm for transient incompressible flow to solve the Navier-Stokes equations. Odours were modeled as passive scalars. To simulate multiple odour sources we found it more efficient to run multiple single-source simulations. As our simulations were deterministic and our scalars passive, this produced equivalent results to simulating multiple sources simultaneously.

The OpenFOAM code for our simulations are provided in the code repository associated with our manuscript at `https://github.com/stootoon/fisher-plumes`.

The plots in Fig S11, Fig S12 and Fig S13 below demonstrate that our results in the Main Text qualitatively hold for this second set of simulations.

S3.2 Testing other probe locations and source geometries

Most of our analyses were performed at a single probe location in each of the two simulations described above. To assess the robustness of those results we extended our analysis to 8 other probe locations in each simulation. We then repeated this procedure for two new arrangements of our sources: one where the sources were placed at 45 degrees to the mean flow direction, and a second where they were parallel to it. The full set of simulations and source arrangements that we used in our work are shown in Fig S10.

S4 Numerical tests of statistical assumptions

Most of the tests below require determining whether two samples of multivariate data come from the same distribution. To determine this, we used the energy test [2]. We computed the energy statistic using Euclidean distance for 1000 permutations of the data and reported the fraction of permutations whose statisticwas higher than what we observed for the unpermuted data. When testing the Gaussianity of a particular sample, we performed the energy test against a randomly generated dataset of the same size and with the same mean and covariance. We averaged the result of three such tests against randomly generated Gaussian data and reported the average p-value.

Location independence. Our first assumption was that the distribution of coefficients from each source was the same for all sources. We tested this by computing p-values of the energy statistic comparing the coefficients from every pair of sources, for each frequency. As shown in Fig S2, these p-values are typically 0.1 or below, consistent with our assumption.

Conditional distribution of coefficients depends only intersource distance. Our second assumption was that the distribution of coefficients from one source, given those at another, depends only on the distance between them. That is, given coefficients (a, b) from source i and (c, d) from source j, with intersource distance D_{ij},

$$p(c, d|a, b, (i, j)) = p(c, d|a, b, D_{ij}).$$

This implies that the conditional distribution of coefficients from pairs of sources the same distance apart are the same,

$$p(c, d|a, b, (i_1, j_1)) = p(c, d|a, b, (i_2, j_2)) \text{ whenever } D_{i_1 j_1} = D_{i_2 j_2}.$$

Rather than testing this relationship for conditional distributions, we tested the relationship for joint distributions

$$p(c, d, a, b|(i_1, j_1)) = p(c, d, a, b|(i_2, j_2)) \text{ whenever } D_{i_1 j_1} = D_{i_2 j_2}.$$

This because the conditional distribution on pairs of sources can be related to the joint distribution as

$$p(c, d|a, b, (i, j)) = \frac{p(c, d, a, b|(i, j))}{p(a, b|i)}.$$

The denominator is the distribution of coefficients from source i. We have previously found that this distribution is largely independent of odour source location so the conditional distribution simplifies to

$$p(c, d|a, b, (i, j)) = \frac{p(c, d, a, b|(i, j))}{p(a, b)}.$$

Therefore, any source dependence comes through through the joint distribution in the numerator.

To test this latter relationship we considered all pairs of sources at a given intersource distance. Each pair yields a joint distribution of coefficients at each frequency. We used the energy test to compare the distribution from one pair at a given frequency with the distribution from every other pair at the same intersource distance and frequency, and repeated this test for every frequency. In Fig S3 we have plotted the p-values of these tests. The figure reveals that the p-values for most of the comparisons are large, consistent with our assumption that the conditional distributions are the same for all pairs of sources at the same intersource distance.

Gaussian coefficients. Our third assumption was that the plumes are Gaussian processes, implying that the distribution of coefficients from each source is bivariate Gaussian. We tested this by using the energy test to compare the coefficients from each source and at each frequency, to an equal number of points randomly generated from a bivariate Gaussian with the same mean and covariance. We repeated this procedure three times and computed the mean of the resulting p-values. In Fig S4 we have plotted these averaged p-values. The white points are the comparisons for which the p-value was 0. The plot reveals that for frequencies above ~ 5 Hz our data is inconsistent with this Gaussianity hypothesis.

Stationarity. Our fourth assumption was that the plumes are temporally stationary. This implies, first, that the distribution of sine from a given source at a given frequency is the same as the distribution of cosine coefficients. To test this we computed the p-values of the energy statistic comparing each such pair of distributions. We have plotted these p-values in Fig S5A, revealing that most values are greater than 0.1, consistent with our hypothesis.

Stationarity also implies that the means of the coefficient distributions at positive frequencies are zero. We tested this using the Wilcoxon signed-rank test for 0 median. We used this Wilcoxon test, which tests the median, not the mean, because it doesn not assume Gaussianity of the data. In Fig S5B,C we have plotted the p-values from this test, revealing that the data is consistent with the coefficients for most sources and frequencies having zero medians. Assuming our coefficients are distributed symmetrically (we did not test this), zero median implies zero mean, consistent with our hypothesis

Finally, stationarity implies that the sine coefficients, a_n, and the cosine coefficients b_n, at each harmonic n are uncorrelated i.e. that the expectation of their product, $\langle a_n b_n \rangle$, is zero. To test this we used the Wilcoxon signed-rank test of zero medians to determine where the product terms $a_n b_n$ have zero median. If the distribution of this product is symmetric (we did not test this), then zero median is equivalent to zero mean. In Fig S5D we have plotted the corresponding p-values for each source and at each frequency. The high p-values we observe reveal that the data are consistent with the product terms having zero median.

Taken together, these test results show that our data are consistent with temporal stationarity.

Conditional Gaussianity. Our final assumption was that the distribution of coefficients at one source, given those at another, are bivariate Gaussian. To test this for a pair of sources (i, j), we took source i to be the one being conditioned upon (the 'conditioning source'). We first assembled the joint data from both sources, consisting of 4-vectors for each of T time windows, where the first two elements of each 4-vector are the coefficients from the first source, and the other two are the corresponding coefficients from the second. To approximately condition the second set of coefficients on the first, we binned the bivariate data from the first source into 9 bins defined by 3 marginal quantiles for each of the two data dimensions. We then pooled the bivariate data from the second source by the bins defined for the first source, and compared each of these pooled data to a bivariate Gaussian distribution using the procedure we have described earlier. If the number of data points in the bin was less than 10, we discarded the values, otherwise we took the average p-value across the 3 trials as the p-value for the corresponding bin. Finally, we averaged these p-values over the 9 bins. In Fig S6 we have plotted the results of comparisons where we conditioned on one source and tested on all those with equal or higher index. The p-values are typically 0.1 or above, consistent with our conditional Gaussian assumption.

S5 Derivations of expectation-maximization updates

We fit models to a set $Y = \{y_1, \ldots, y_N\}$ of observed correlations at a given harmonic and intersource distance. The parameters for each model can be written as $\theta = \{\iota, \sigma, \theta^+, \theta^-\}$. The intermittency level ι and noise distribution standard deviation σ, are common to all the models. The remaining parameters, grouped into two sets θ^+ and θ^-, are specific to each model and are used to fit the positive and negative correlations, respectively.

To fit model parameters θ to the correlation data Y, we assumed that the observations they were independent and identically distributed according to Eqn 95 above. We then used maximum-*a posteriori* (MAP)

estimation to estimate the values of the model parameters. That is, we maximized

$$\log p(\theta|Y) \doteq \log p(\theta) + \log p(Y|\theta) = \log p(\theta) + \sum_{i=1}^{M} \log p(y_i|\theta) \tag{S6}$$

{log_ptheta}

over θ. The dot over the first equal sign indicates that the equality is up to the additive constant, namely $-\log p(Y)$ which does not depend on θ. The expansion into the sum in the last term is due to our assumption of independence of the observations.

To maximize Eqn S6 for θ we must account for the variables $z_1 \dots z_N$ upon which the observations depend. Because these variables are latent, we use the expectation maximization algorithm [3] to iteratively maximize a lower bound to Eqn S6. At each step of the iteration, we estimate the values of the latent variables $Z = \{z_1, \dots, z_N\}$ (the E-step), and then use those values to estimate the parameters θ of the model. We iterate until the change in parameters is less than a predefined tolerance.

Expectation maximization can be seen as coordinate ascent on the *negative variational free energy* [4]. This quantity relates a distribution $q(Z|Y, \theta)$ on the latents to their posterior distribution $p(Z|Y, \theta)$. It has two equivalent forms

$$F(q, \theta) = \log p(Y|\theta) - D_{\text{KL}}(q(Z|Y, \theta)||p(Z|Y, \theta)) \tag{S7a}$$

{F_E}

$$= \mathbb{E}_q \log p(Y, Z|\theta) + H(q), \tag{S7b}$$

{F_M}

where $H(q)$ is the entropy of the proposed distribution. From Eqn S7a and the non-negativity of the KL divergence we can see that the free energy is a lower bound on the log likelihood. During the E-step, we maximize this lower bound with respect to q. During the M-step, we use the form of the free energy in Eqn S7b to maximize it with respect to θ.

To perform expectation maximization we require a distribution $q(Z|Y, \theta)$ to model the posterior probability of the latent variables. We will assume that this distribution factorizes, so that

$$q(Z|Y, \theta) = \prod_{i=1}^{N} q(z_i|Y, \theta). \tag{S8}$$

{qZ_fact}

To specify $q(z_i|Y, \theta)$ we will use a distribution that puts all the probability mass for each variable on the most likely value, whether 0 or 1.

S5.1 E-step

Maximizing Eqn S7a with respect to q is equivalent to minimizing $D_{\text{KL}}(q(Z|Y, \theta)||p(Z|Y, \theta))$. We can write this as

$$\begin{aligned} D_{\text{KL}}(q(Z|Y, \theta)||p(Z|Y, \theta)) &= \sum_Z q(Z|Y, \theta) \log \frac{q(Z|Y, \theta)}{p(Z|Y, \theta)} = \sum_Z q(Z|Y, \theta) \log \frac{q(Z|Y, \theta)P(Y|\theta)}{p(Y|Z, \theta)P(Z|\theta)} \\ &= -\sum_{i=1}^{N} \left[H(q(z_i)) + \sum_{z_i \in \{0,1\}} q(z_i) \log p(y_i|z_i, \theta) p(z_i|\theta) \right], \end{aligned} \tag{S9}$$

where we've shortened $q(z_i|Y, \theta)$ to $q(z_i)$. Minimizing this term over z_i means maximizing each of the terms in square brackets. The entropy terms are all zero because q puts all the probability mass on zero or one. To determine whether z_i should be set to zero or one, we maximize the second term, which yields

$$z_i = \begin{cases} 1 & \text{if } \log \frac{p(y_i|z_i=1,\theta)p(z_i=1|\theta)}{p(y_i|z_i=0,\theta)p(z_i=0|\theta)} = \log \frac{\rho(y_i|\theta)\iota}{\eta(y_i|\theta)(1-\iota)} > 0, \\ 0 & \text{otherwise.} \end{cases} \tag{S10}$$

After rearranging, this is Eqn 38 in the Main Text.

S5.2 M-step

During the M-step we maximize Eqn S7b plus the log prior with respect to the parameters θ. For our model the entropy term is always zero so we need only maximize the joint distribution of observations and latents given the parameters. Also, we only use a prior distribution for the intermittency parameter ι. So,

$$\theta_{t+1} = \max_{\theta} \; J(\theta) \tag{S11}$$
{max_J}

$$J(\theta) \triangleq \mathbb{E}_q \log p(Y, Z|\theta) = \log p(\iota) + \mathbb{E}_{q(Z)} \log p(Y|Z, \theta)p(Z|\theta) = \log p(\iota) + \sum_{i=1}^{N} \mathbb{E}_{q(z_i)} \log p(y_i|z_i, \theta)p(z_i|\theta). \tag{S12}$$
{J_theta}

We can split the sum into components over those observations marked as correlations in the E-step, indexed by the set I^1, and those marked as noise, indexed as I^0:

$$\begin{aligned} J(\theta) &= \log p(\iota) + \sum_{i \in I^0} (\log(1-\iota) + \log \eta(y_i|\sigma)) + \sum_{i \in I^1} (\log \iota + \log \rho(y_i|\theta)) \\ &= \log p(\iota) + N^0 \log(1-\iota) + \sum_{i \in I^0} \log \eta(y_i|\sigma) + N^1 \log \iota + \sum_{i \in I^1} (\log \rho(y_i|\theta), \end{aligned} \tag{S13}$$

where $N^0 = |I^0|$ is the number labeled as noise, and $N^1 = |I^1|$ is the number of observations labeled as correlations. We can split this further by the sign of the correlations

$$J(\theta) = \log p(\iota) + N^0 \log(1-\iota) + \sum_{i \in I^0} \log \eta(y_i|\sigma) + N^1 \log \iota + \sum_{i \in I^+} \log \rho(y_i|\theta^+) + \sum_{i \in I^-} \log \rho(y_i|\theta^-), \tag{S14}$$
{J_final}

where $I^+ \subset I^1$ indexes the subset of the correlations that are positive, and $I^{-1} \subset I^1$ indexes the subset that are negative. Letting $N^+ = |I^+|$ and $N^- = |I^-|$ be the sizes of these sets, we have $N^+ + N^- = N^1$.

We now maximize Eqn S14 with respect to the parameters.

S5.2.1 Intermittency parameters

ι update. We put a Beta prior on ι with mean and sample size hyperparameters, respectively, so that

$$p(\iota) \propto \iota^{\mu\nu}(1-\iota)^{(1-\mu)\nu}. \tag{S15}$$
{beta_prior}

Then setting the gradient of $J(\theta)$ with respect to ι to zero gives

$$\frac{\partial J}{\partial \iota} = 0 = \frac{\mu\nu}{\iota} + \frac{(1-\mu)\nu}{1-\iota} + \frac{N^1}{\iota} - \frac{N^0}{1-\iota} = \frac{\mu\nu + N^1}{\iota} - \frac{(1-\mu)\nu + N^0}{1-\iota},$$

Solving for ι yields

$$\iota = \frac{N^1 + \mu\nu}{N + \nu}.$$

Substituting $\bar{\iota}_0$ for μ and $N\alpha$ for ν gives Eqn 40

σ^2 update. To update the standard deviation σ of the noise distribution we compute

$$\frac{\partial J}{\partial \sigma} = -\frac{\partial}{\partial \sigma} \sum_{i \in I^0} \frac{1}{2} \log(2\pi\sigma^2) + \frac{y_i}{2\sigma^2} = \sum_{i \in I^0} \frac{1}{\sigma} - \frac{y_i^2}{\sigma^3} = 0. \tag{S16}$$
{sig_grad}

Solving for σ^2 yields Eqn 41.

S5.2.2 Correlation parameters

From Eqn S14, maximizing the correlation parameter θ^+ and θ^- can be done separately by maximizing the likelihood of the positive, and negative observations, respectively, that were marked as correlations in the E-step. This was done analytically for the Exponential model and numerically for the remaining two models.

Exponential model. The Exponential model parameters are $\theta^+ = \lambda$ and $\theta^- = \mu$, with correlation distribution

$$\rho(y|\theta) = \frac{1}{\lambda+\mu} \begin{cases} \exp(-|y|/\lambda) & y \geq 0 \\ \exp(-|y|/\mu) & y < 0. \end{cases} \tag{S17}$$ {exp_corr}

To determine λ, we consider a positive correlation $y_i > 0$. Then

$$\log \rho(y_i|\theta) = -\log(\lambda+\mu) - \frac{|y_i|}{\lambda}. \tag{S18}$$ {logrho}

Taking the gradient of Eqn S14,

$$\frac{\partial J}{\partial \lambda} = \sum_{i\in I^1} -\frac{1}{\lambda+\mu} + \sum_{i \in I^+} \frac{|y_i|}{\lambda^2} = -\frac{N}{\lambda+\mu} + \frac{\sum_{i\in I^+}|y_i|}{\lambda^2} \propto -\frac{1}{\lambda+\mu} + \frac{N^+}{N}\frac{\overline{|y_+|}}{\lambda^2}. \tag{S19}$$ {dladt}

Here $\overline{|y_+|}$ is the average positive correlation. Similarly,

$$\frac{\partial J}{\partial \mu} \propto -\frac{1}{\lambda+\mu} + \frac{N^-}{N}\frac{\overline{|y_-|}}{\mu^2}, \tag{S20}$$ {dmudt}

where $\overline{|y_i|}$ is the average negative correlation. Setting Eqn S19 and Eqn S20 to zero, we get

$$\frac{N^+}{N}\overline{|y_+|} = \frac{\lambda^2}{\lambda+\mu}, \quad \frac{N^-}{N}\overline{|y_-|} = \frac{\mu^2}{\lambda+\mu}. \tag{S21}$$

Solving for λ and μ gives the updates in Eqn 42

Gamma model. The model parameters are $\theta^+ = \{\lambda, k\}$ and $\theta^- = \{\mu, m\}$, with correlation distribution

$$\rho(y|\theta) = \frac{1}{\Gamma(m)\mu^m + \Gamma(k)\lambda^k} \begin{cases} |y|^{k-1}\exp(-|y|/\lambda) & y \geq 0 \\ |y|^{m-1}\exp(-|y|/\mu) & y < 0. \end{cases} \tag{S22}$$ {gam_corr}

Taking the negative logarithm and summing over correlations by sign gives Eqn 43b, which we numerically minimize.

Generalized inverse Gaussian model. The model parameters are $\theta^+ = \{\lambda, k, \alpha\}$ and $\theta^- = \{\mu, m, \beta\}$, with correlation distribution

$$\rho(y|\theta) = \frac{1}{2}\frac{1}{\lambda K_v(k,\alpha) + \mu K_v(m\beta)} \begin{cases} \left(\frac{|y|}{\lambda}\right)^{k-1} \exp\left(-\frac{\alpha}{2}\left[\frac{|y|}{\lambda} + \frac{\lambda}{|y|}\right]\right) & y \geq 0, \\ \left(\frac{|y|}{\mu}\right)^{m-1} \exp\left(-\frac{\beta}{2}\left[\frac{|y|}{\mu} + \frac{\mu}{|y|}\right]\right) & y < 0, \end{cases} \tag{S23}$$ {gam_corr}

where $K_v(a,b)$ is the modified Bessel function of the second kind of real order a evaluated at b. We use the negative logarithm of this distribution, summed over correlations by sign, in the damped update of Eqn 44a.

S6 Miscellaneous

S6.1 Implications of temporal stationarity

Our assumption that odour concentration profiles are temporally stationary has several important implications. First, it implies that the marginal distribution of the sine and cosine coefficients must be the same,

$$p(a_n = v) = p(b_n = v). \tag{S24}$$ {marg_coef}

To see why, consider computing coefficients a_n and b_n at two time points $t_2 > t_1$ separated by one-quarter the period of the n'th harmonic. Note that a sine waveform starting at the earlier time point becomes a cosine waveform at the later one. This means that the statistics of the sine coefficients at the earlier time point are the same as those of the cosine coefficients at the later time point, that is $p(b_n|_{t=t_1}) = p(a_n|_{t=t_2})$. By stationarity we know that the statistics of the sine coefficients at the two time points are the same, that is $p(b_n|_{t=t_1}) = p(b_n|_{t=t_2})$. Combining these equalities yields Eqn S24.

A second implication is that the coefficients at non-zero frequency have mean zero. Intuitively, this is because any other value would introduce a non-stationarity into the plume signal that could not be canceled by any combination of the other harmonics due to their linear independence. Formally, by the linearity of Fourier decomposition the coefficients for the mean plume signal are the means of the coefficients for the plume. By stationarity, the mean plume signal is constant in time. Therefore, the mean value of the coefficients at non-zero frequencies must be zero. That is,

$$\langle a_{n\neq 0}\rangle = \langle b_{n\neq 0}\rangle = 0, \tag{S25}$$

where expectations are over time windows.

A third implication is that the sine and cosine coefficients are uncorrelated. Intuitively, this is because the sine and cosine coefficients together determine the phase of the sinusoid at a given harmonic, and any correlation between the coefficients would produce non-stationarity in the phase distribution of the signal. Formally, consider computing correlations at time points $t_2 > t_1$ separated by a quarter period as before. As we previously noted, a sine waveform at t_1 becomes a cosine waveform at t_2. A cosine waveform at t_1, however, becomes a <u>minus</u> sine at t_2. Therefore b_n at t_1 maps to a_n at t_2, while a_n at t_1 maps to $-b_n$ at t_2. We thus have

$$\langle a_n b_n\rangle|_{t_1} = \langle(-b_n)a_n\rangle|_{t_2} = -\langle a_n b_n\rangle|_{t_2}, \tag{S26}$$

{zero_corr1

where the expectations are over $t_2 > t_1$ pairs separated by a quarter cycle. By stationarity the statistics at t_1 and t_2 must be the same. Therefore,

$$-\langle a_n b_n\rangle|_{t_2} = \langle a_n b_n\rangle|_{t_2}, \tag{S27}$$

{zero_corr2

and we arrive at

$$\langle a_n b_n\rangle = 0 \tag{S28}$$

where expectation is over time. Related to this point, a final implication involves the correlations of coefficients from different sources. Applying the same considerations of the two time points t_1 and t_2 to the coefficients c_n and d_n from a second source, we find that

$$\langle a_n c_n\rangle = \langle b_n d_n\rangle, \tag{S29}$$

{ac_bd}

and

$$\langle a_n d_n\rangle = \langle b_n, -c_n\rangle = -\langle b_n c_n\rangle. \tag{S30}$$

{ad_bc}

S6.2 Residual coefficients are uncorrelated

The correlation of the residual coefficients in Eqn 66 is

$$\begin{aligned}
\langle\tilde{c}_n, \tilde{d}_n\rangle &= \langle c_n, d_n\rangle && \text{(S31a)} \quad \{t1\}\\
&- \langle d_n\beta_n(a_n\cos(\theta_n) + b_n\sin(\theta_n))\rangle && \text{(S31b)} \quad \{t2\}\\
&- \langle c_n\beta_n(b_n\cos(\theta_n) - a_n\sin(\theta_n))\rangle && \text{(S31c)} \quad \{t3\}\\
&+ \langle\beta_n^2(a_n\cos(\theta_n) + b_n\sin(\theta_n))(b_n\cos(\theta_n) - a_n\sin(\theta_n))\rangle && \text{(S31d)} \quad \{t4\}
\end{aligned}$$

To see that this correlation is zero, note first that the right-hand side of Eqn S31a is zero since c_n and d_n are uncorrelated (Eqn 9). Next, dropping the β_n^2 term from Eqn S31d and collecting terms we have

$$\langle a_n b_n\rangle\cos(\theta_n)^2 - \langle a_n b_n\rangle\sin(\theta_n)^2 + (\langle b_n^2\rangle - \langle a_n^2\rangle)\sin(\theta_n)\cos(\theta_n) = 0 \tag{S32}$$

since a_n and b_n are uncorrelated, and have the same variance. Finally, combining Eqn S31b and Eqn S31c and dropping β_n, we have

$$-\langle a_n d_n + b_n c_n \rangle \cos(\theta_n) + \langle a_n c_n - b_n d_n \rangle \sin(\theta_n) = 0 \tag{S33}$$

by the implications of temporal stationarity in Eqn S29 and Eqn S30. Therefore, the terms in Eqn S31 are all either individually or in combination zero, and we conclude that their sum is zero.

S6.3 Fits at very low frequencies

The data in Fig 7G suggests that the fits at 1 Hz are poor. We've highlighted those fits in Fig S15 below. These poor fits are likely due to the window size, as expanding it to 2 seconds improves the 1 Hz fits; see Fig S16 below.

S6.4 Converting Fourier coefficients to trigonometric coefficients

The STFT performed on plumes provides (discrete) Fourier transform coefficients. To convert the Fourier coefficients $X[k]$ of a length L signal $x[n]$ to the equivalent trigonometric representation we used the fact that the (discrete) Fourier transform of a real signal of length L satisfies $X[k] = \overline{X[L-k]}$, where the bar indicates complex conjugation. If we write each Fourier coefficient as $X[k] = u_k + jv_k$ where j is the imaginary unit, we have

$$x[n] = X[0] + \sum_{k=1}^{L-1} X[k] e^{j2\pi kn/L} = X[0] + \sum_{k=1}^{L-1} (u_k + jv_k)\cos(2\pi kn/L) + (ju_k - v_k)\sin(2\pi kn/L).$$

Then, if the signal is of odd length, we have

$$\begin{aligned} x[n] &= X[0] + \sum_{k=1}^{(L-1)/2} ((u_k + jv_k) + (u_k - jv_k))\cos(2\pi kn/L) + ((ju_k - v_k) + (-ju_k - v_k))\sin(2\pi kn/L) \\ &= X[0] + \sum_{k=1}^{(L-1)/2} 2u_k \cos(2\pi kn/L) - 2v_k \sin(2\pi kn/L). \end{aligned} \tag{S34}$$

{oddsig}

If the signal is of even length, we have

$$\begin{aligned} x[n] &= X[0] + \left(\sum_{k=1}^{\lfloor \frac{L-1}{2} \rfloor} ((u_k + jv_k) + (u_k - jv_k))\cos(2\pi kn/L) + ((ju_k - v_k) + (-ju_k - v_k))\sin(2\pi kn/L) \right) \\ &+ (u_{L/2} + jv_{L/2})\cos(\pi n) \\ &= X[0] + u_{L/2}\cos(\pi n) + \sum_{k=1}^{\lfloor \frac{L-1}{2} \rfloor} 2u_k \cos(2\pi kn/L) - 2v_k \sin(2\pi kn/L), \end{aligned} \tag{S35}$$

{evensig}

where $\lfloor \cdot \rfloor$ is the floor function. The last equality holds because $v_{L/2} = 0$ since $X[L/2]$ is the correlation of the signal with $\exp(j\pi n) = \cos(\pi n)$, which is purely real. We can then combined Eqn S34 and Eqn S35 to write the signal in terms of sine and cosine coefficients as in Eqn 27.

S6.5 Effect of windowing on covariances

We will perform the computation in continuous time for simplicity. Let two plumes generated at locations p and q be $x_p(t)$ and $x_q(t)$. We will take the full duration of these plumes to be the time interval $[0, T]$, where we'll set $T = 1$ for convenience. Performing Fourier decompositions we have

$$x_p(t) = \sum_{k=0}^{\infty} X_p[k] \exp(j2\pi kt), \quad x_q(t) = \sum_{k=0}^{\infty} X_q[k] \exp(j2\pi kt). \tag{S36}$$

{xab}

Plumes are stochastic so $X_p[n]$ and $X_q[n]$ are random variables. The covariance kernel $K(p, q, n)$ determines how these variables are related,

$$K(p, q, n) = \mathbb{E}(X_p[n], \overline{X_q[n]}), \tag{S37}$$

{kerker}

where the expectation is taken over time windows and the overline means complex conjugation. For convenience we will use this definition of the covariance kernel, relating complex Fourier coefficients, rather than Eqn 45 used in the Main Text, relating the expression of the Fourier coefficients as trigonometric functions.

The animal is unable to observe the entire plume and only sees a windowed version of it. Using tilde to indicate a windowed quantity, the animal observes the plume from location p as

$$\widetilde{x}_p(t) = x_p(t)w(t) \tag{S38}$$

where $w(t)$ is the windowing function that truncates the plume to a smaller time window, applies exponential filtering etc. Windowing a signal convolves its spectrum with that of the window,

$$\tilde{X}_p[k] = (X_p * W)[k] \tag{S39}$$

making the resulting kernel

$$\begin{aligned}
\widetilde{K}(a, b, k) &= \mathbb{E}\left\{(X_p * W)[k]\overline{(X_q * W)}[k]\right\} \\
&= \mathbb{E}\left\{\sum_{m,m'} (X_p[m]\overline{W[k-m]})(\overline{X_q[m']}W[k-m'])\right\} \\
&= \sum_{m,m'} \mathbb{E}\left\{X_p[m]\overline{X_q[m']}\right\} \overline{W[k-m]}W[k-m'].
\end{aligned}$$

The expectation in the expression above is the covariance of coefficients from plumes at two different locations for two frequency components k and k'. When comparing the coefficients of a plume to itself, the covariance of coefficients at different frequency components will tend towards 0 if the interval over which the coefficients are computed is large enough [5]. We will assume that this condition is satisfied. Since the covariance between plumes from different sources will be weaker than that of a plume with itself, we can take this covariance to be 0 when comparing different frequency components. That is,

$$\mathbb{E}\left\{X_p[m]\overline{X_q[m']}\right\} \approx \mathbb{E}\left\{X_p[m]\overline{X_q[m]}\right\} \delta_{m,m'} = K(p, q, m)\delta_{m,m'}. \tag{S40}$$

We then have

$$\begin{aligned}
\widetilde{K}(a, b, k) &\approx \sum_{m,m'} K(a, b, m)\delta_{m,m'}\overline{W[k-m]}W[k-m'] \\
&= \sum_{m} K(a, b, m)W[k-m]^2.
\end{aligned} \tag{S41}$$

{kerconv}

Thus the kernel $\widetilde{K}$ of the windowed signal is approximately the convolution of the true kernel K with the squared amplitude spectrum of the windowing function. Since the relevant timescale here is that of an entire plume (10s of seconds), rather than the window (O(1 second)), the approximation is likely to be very good in practice and we will treat the approximate equality as an equality in what follows.

If the true kernel is decomposable as a product of a spatial and a frequency kernel,

$$K(a, b, m) = G(a, b)S(m) \tag{S42}$$

{kersep1}

then the windowed kernel is also decomposable

$$\widetilde{K}(a, b, k) = \sum_{k} G(a, b)S(m)W[k-m]^2 = G(a, b)\sum_{k} S(m)W[k-m]^2. \tag{S43}$$

{kerwindec}

From Eqn 23 the spatial information in the kernel is related to its derivative with respect to the spatial separation $|a-b|$, which is

$$\frac{\partial \widetilde{K}(a,b,k)}{\partial |a-b|} = \frac{\partial G(a,b)}{\partial |a-b|} \sum_k S(m) W[k-m]^2. \tag{S44}$$

The effect of the window is thus a scaling of the kernel by the convolution of the frequency kernel $S(m)$ with the squared spectrum of the window. Since the information content only depends on the shape of the kernel as a function of distance and not its absolute scaling, the spatial information in the windowed kernel is the same as in the un-windowed case.

If, however, the kernel is not decomposable as Eqn S42, then its shape, and thus its information content, can be changed by windowing. For example, consider a kernel which exhibits spatial decay with space-constant L_0 for all frequencies components except at k_1, for which the space constant is L_1:

$$K(a,b,k) = S(k) \exp\left(-\frac{|a-b|}{L[k]}\right), \quad L[k] = \begin{cases} L_1 & \text{if } k = k_1, \\ L_0 & \text{otherwise.} \end{cases} \tag{S45}$$

The windowed kernel is

$$\widetilde{K}(a,b,k) = \sum_m W[k-m]^2 S(m) \exp\left(-\frac{|a-b|}{L[m]}\right). \tag{S46}$$

The derivative with respect to the spatial separation $|a-b|$ is

$$\begin{aligned} \frac{\partial \widetilde{K}(a,b,k)}{\partial |a-b|} &= -\sum_m \frac{W[k-m]^2 S(m)}{L[m]} \exp\left(-\frac{|a-b|}{L[m]}\right) \\ &= -\frac{W[k-k_1]^2 S(m_1)}{L_1} \exp\left(-\frac{|a-b|}{L_1}\right) - \sum_{m \neq k_1} \frac{W[k-m]^2 S(m)}{L_0} \exp\left(-\frac{|a-b|}{L_0}\right) \end{aligned} \tag{S47}$$

By adding and subtracting terms we can write this as a correction added to the derivative of the windowed kernel that has a single space constant L_0

$$\begin{aligned} \frac{\partial \widetilde{K}(a,b,k)}{\partial |a-b|} &= -\left[\sum_m W[k-m]^2 S(m)\right] \frac{1}{L_0} \exp\left(-\frac{|a-b|}{L_0}\right) \\ &\quad + W[k-k_1]^2 S(k_1) \left[\frac{1}{L_0} \exp\left(-\frac{|a-b|}{L_0}\right) - \frac{1}{L_1} \exp\left(-\frac{|a-b|}{L_1}\right)\right]. \end{aligned} \tag{S48}$$

{K1}

If, for example, the power $S(k_1)$ at the frequency component with the space-constant L_1 is sufficiently low, then the correction term will be dominated by the derivative of the kernel with constant space constant. To see this, we can evaluate the derivative in Eqn S48 at $k = k_1$. Doing so for the true kernel we get,

$$\left.\frac{\partial K(a,b,k)}{\partial |a-b|}\right|_{k=k_1} = -S(k_1) \frac{1}{L_1} \exp\left(-\frac{|a-b|}{L_1}\right), \tag{S49}$$

{dKtrue}

where we've assumed $W[0]^2 = 1$. For the windowed kernel,

$$\left.\frac{\partial \widetilde{K}(a,b,k)}{\partial |a-b|}\right|_{k=k_1} = -S(k_1) \frac{1}{L_1} \exp\left(-\frac{|a-b|}{L_1}\right) - \left[\sum_{m \neq k_1} W[k_1-m]^2 S(m)\right] \frac{1}{L_0} \exp\left(-\frac{|a-b|}{L_0}\right). \tag{S50}$$

{dKwin}

This is just the value for the true kernel in Eqn S49 corrupted by the values for neighbouring frequencies through the spectral leakage (the term in square brackets).

Thus when the true kernel is not decomposable as a product of space-only and frequency-only parts, the spatial information available in the windowed plume can differ from that in the full plume. This can result in frequencies that are particularly informative in the true signal being masked by spectral leakage in the windowed signal.

S6.6 Relating covariance kernels to Fisher information

How does the kernel $K(n,s)$ used to generate a surrogate dataset determine Fisher information? In Eqn 23 we have expressed it in terms of the average in-phase correlation $\rho_n(s)$. To express Fisher information in terms of the covariance kernel $K(n,s)$, we multiply the numerator and denominator by σ^4, to get

$$\mathcal{I}(r_n, s) = \frac{1}{\sigma^4 - \sigma^4 \rho_n(s)^2} \left[\frac{d\sigma^2 \rho_n(s)}{ds} \right]^2 . \tag{S51}$$

{fisherker0

The variance corresponds to the value of the kernel at an intersource separation of 0. Therefore,

$$K(n, 0) = \sigma_n^2. \tag{S52}$$

{kvar0}

The values of the kernel at $s > 0$ determine the covariance of coefficients from different sources. Therefore, from Eqn 14b we have that

$$K(n, s) = \sigma^2 \rho_n(s) \tag{S53}$$

{kvar}

Substituting Eqn S52 and Eqn S53 into Eqn S51 gives the Fisher information interms of the kernels as

$$\mathcal{I}(r_n, s) = \frac{1}{K(n,0)^2 - K(n,s)^2} \left[\frac{dK(n,s)}{ds} \right]^2 . \tag{S54}$$

{fisherker}

Thus we can change the amount of spatial information present in the correlations by changing the shape of the kernel, as expected. For example, we can consider a kernel that does not change with intersource distance. In that case, $dK(n,s)/ds = 0$. Substituting this into Eqn S54 (taking appropriate care of the zero in the denominator) reveals the Fisher information to be zero, as expected.

A more interesting kernel is one that can be decomposed into a product of a term $S(n)$ that depends only on the frequency component n, and a term $G(s)$ that depends only on the spatial separation:

$$K(n, s) = S(n) G(s). \tag{S55}$$

{sepker}

The first term in the product, $S(n)$, reflects the variance of the coefficients of the n'th frequency component. The second term, $G(s)$, reflects the correlations at intersource separation s. Because this term is not a function of the frequency component n, correlations at all frequencies change the same way with intersource separation, as observed in Fig 4F. For such 'separable' kernels, all frequencies are equally informative. Indeed, substituting Eqn S55 into our new expression for Fisher information in Eqn S54 yields

$$\mathcal{I}(r_n, s) = \frac{1}{1 - G(s)^2} \left[\frac{dG(s)}{ds} \right]^2 , \tag{S56}$$

{Isep}

which depends only on the intersource separation s, and not the harmonic n.

References

[1] H. G. Weller, G. Tabor, H. Jasak, and C. Fureby. A tensorial approach to computational continuum mechanics using object-oriented techniques. Computers in Physics, 12(6):620–631, November 1998. ISSN 0894-1866. doi: 10.1063/1.168744. URL https://aip.scitation.org/doi/abs/10.1063/1.168744. Publisher: American Institute of Physics.

[2] Gabor J Szekely and Maria L Rizzo. Testing for equal distributions in high dimension. 2004.

[3] Christopher M. Bishop. Pattern Recognition and Machine Learning. Springer, 2006.

[4] D. Koller and N. Friedman. Probabilistic Graphical Models: Principles and Techniques. MIT Press, 2009.

[5] Nelson M. Blachman. On fourier series for Gaussian noise. Information and Control, 1(1):56–63, September 1957.
