# Supplementary figures and images for: Quantifying spectral information about source separation in multisource odour plumes
Source: PLoS One. 2025 Jan 10;20(1):e0297754. doi: 10.1371/journal.pone.0297754 (PMC11723556; doi:10.1371/journal.pone.0297754)

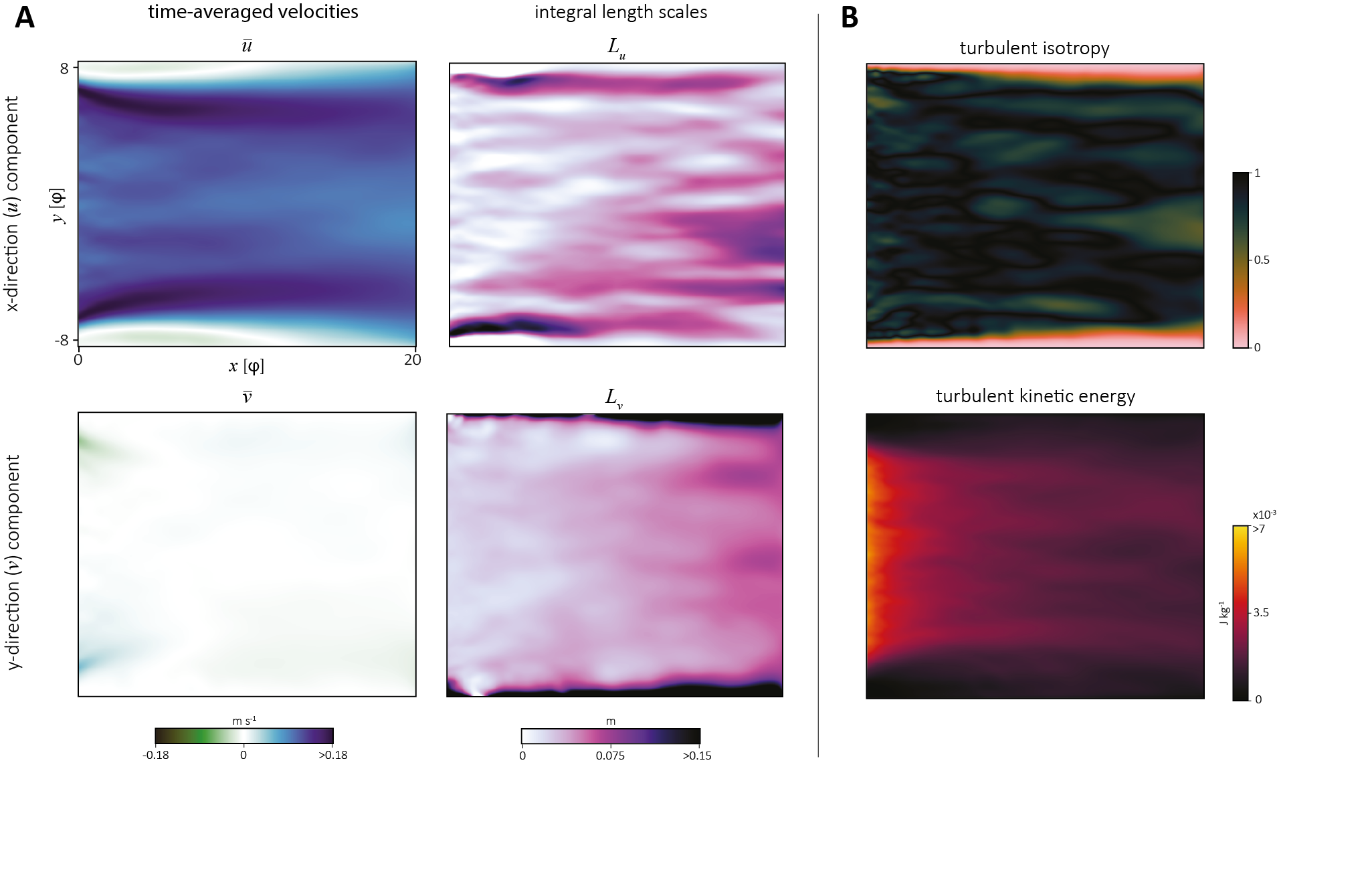

Supplement: S1 Fig — (A) horizontal (top) and vertical (bottom) components of the mean velocity (left) and integral length scale (right) over the simulated plume domain. (B) mean turbulent isotropy (top) and turbulent kinetic energy (bottom) over the simulated plume domain. (TIF) [file pone.0297754.s002.tif]

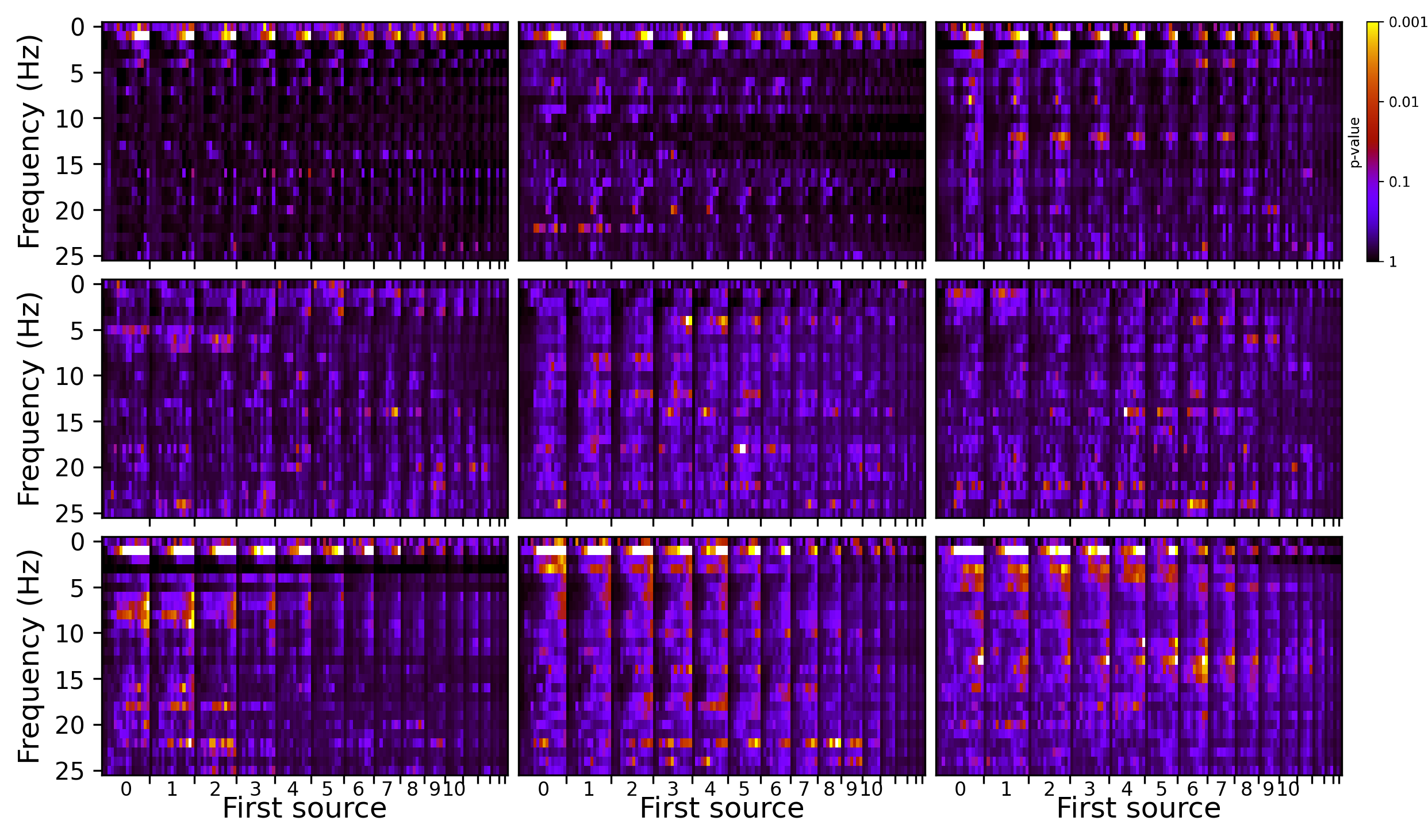

Supplement: S2 Fig — p-values of the energy test measuring the difference between the distribution of coefficients at one source (‘First source’) and those at another, at each frequency. Each column corresponds to the comparison for one pair of sources. (TIF) [file pone.0297754.s003.tif]

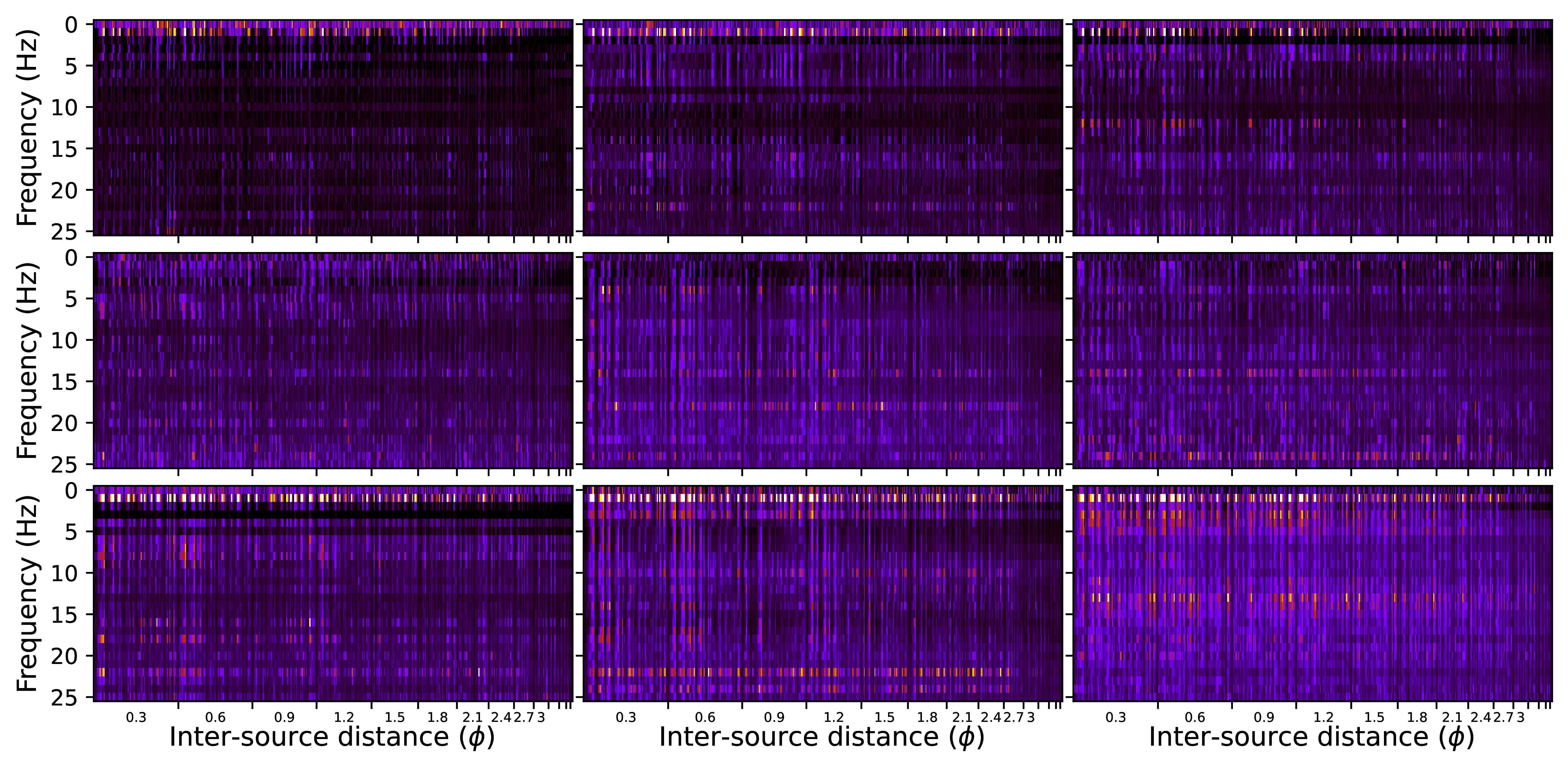

Supplement: S3 Fig — p-values of the energy test measuring the difference between the distributions of coefficients from all pairs of sources at a given intersouce distance (indicated on the x-axis), for each frequency. Each column corresponds to the comparison of one pair of sources. There were multiple pairs of odour sources at each intersource distance, indicated by the sizes of each block. Colours as in S2 Fig. (TIF) [file pone.0297754.s004.tif]

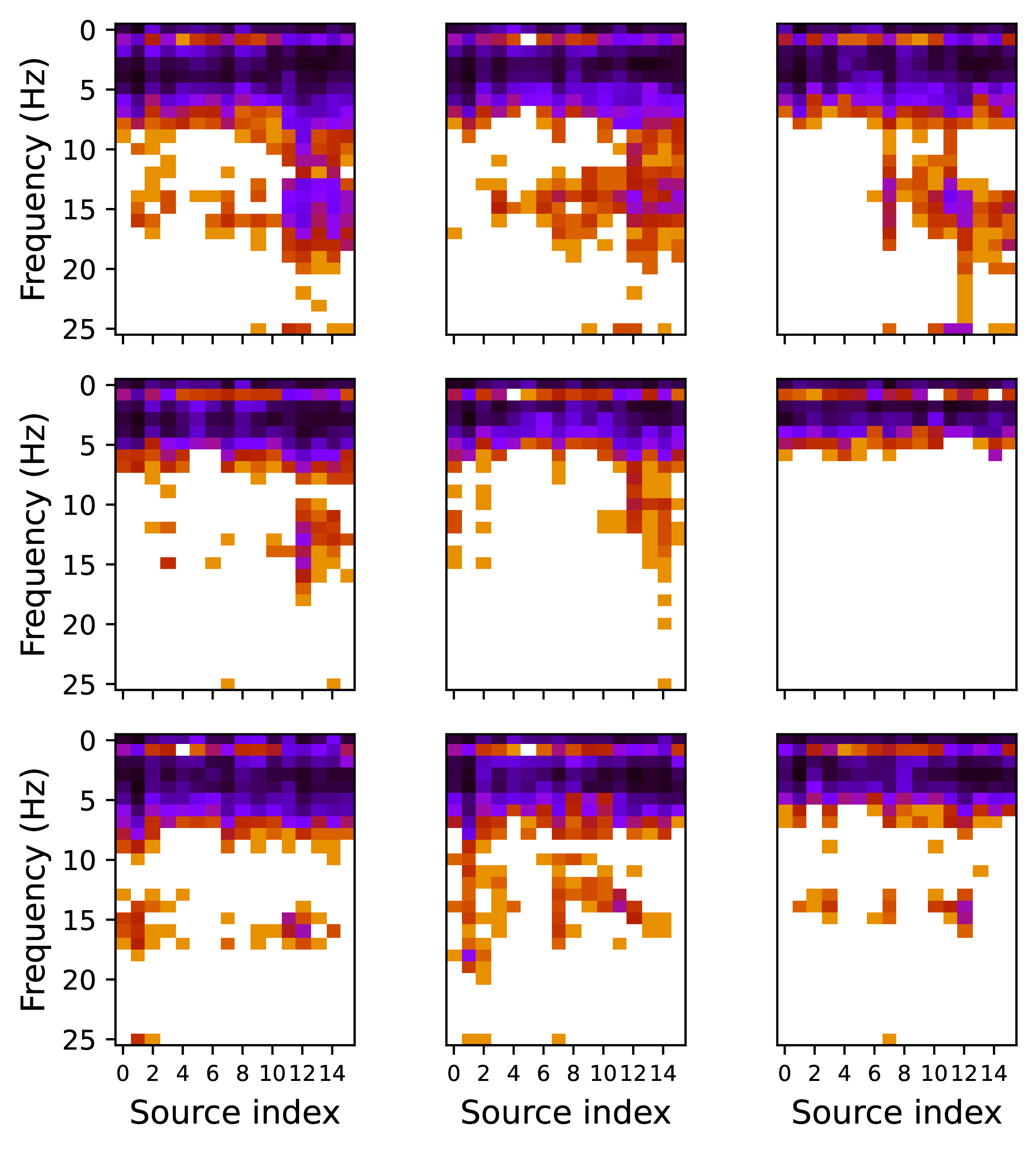

Supplement: S4 Fig — p-values of the energy test measuring the difference between the distribution of coefficients at each source to those of a bivariate Gaussian with the same mean and covariance, at each frequency. Colours as in S2 Fig, white indicates p < 0.001. (TIF) [file pone.0297754.s005.tif]

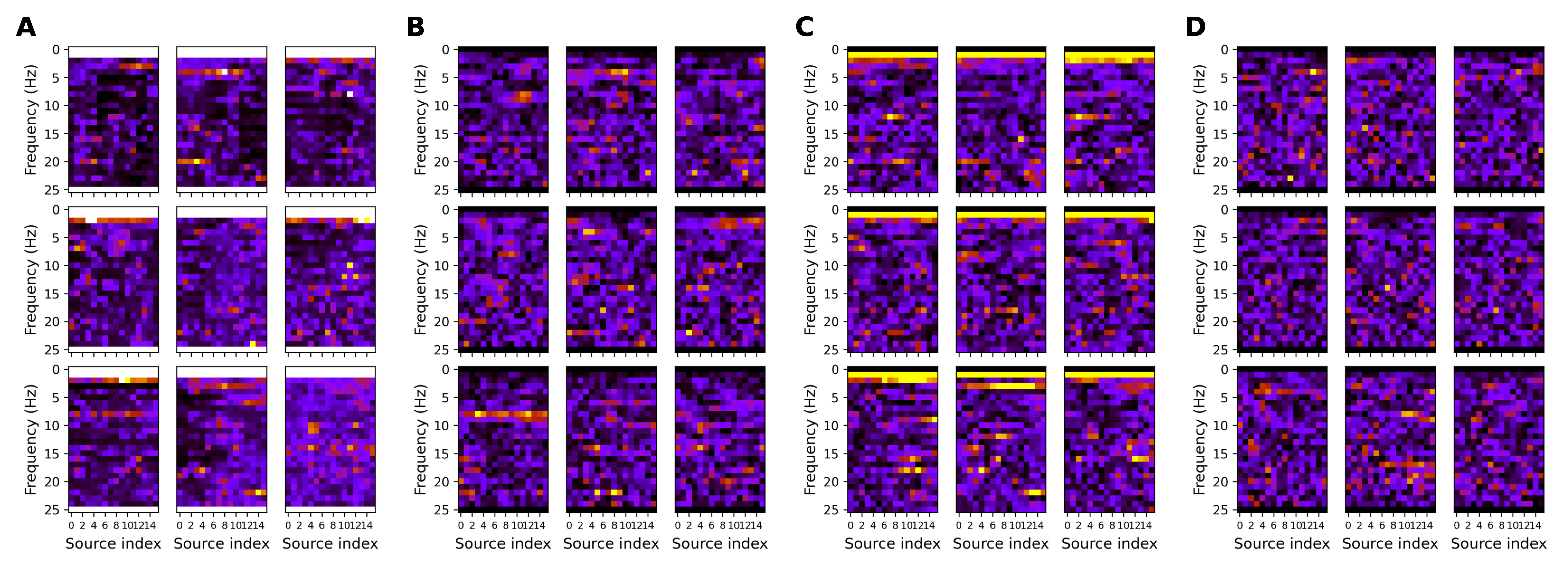

Supplement: S5 Fig — (A) p-values for the energy test comparing the distribution of sine coefficients to that of the cosine coefficients, at each source and frequency. (B,C) p-values of the Wilcoxon signed-rank test for whether the medians of the distributions of the sine, and cosine, coefficients is zero, for the data from each each source and each frequency. (D) p-values of the Wilcoxon signed-rank test for whether the median of the product of the sine and cosine coefficients is zero. Colours as in S2 Fig, white indicates p < 0.001. (TIF) [file pone.0297754.s006.tif]

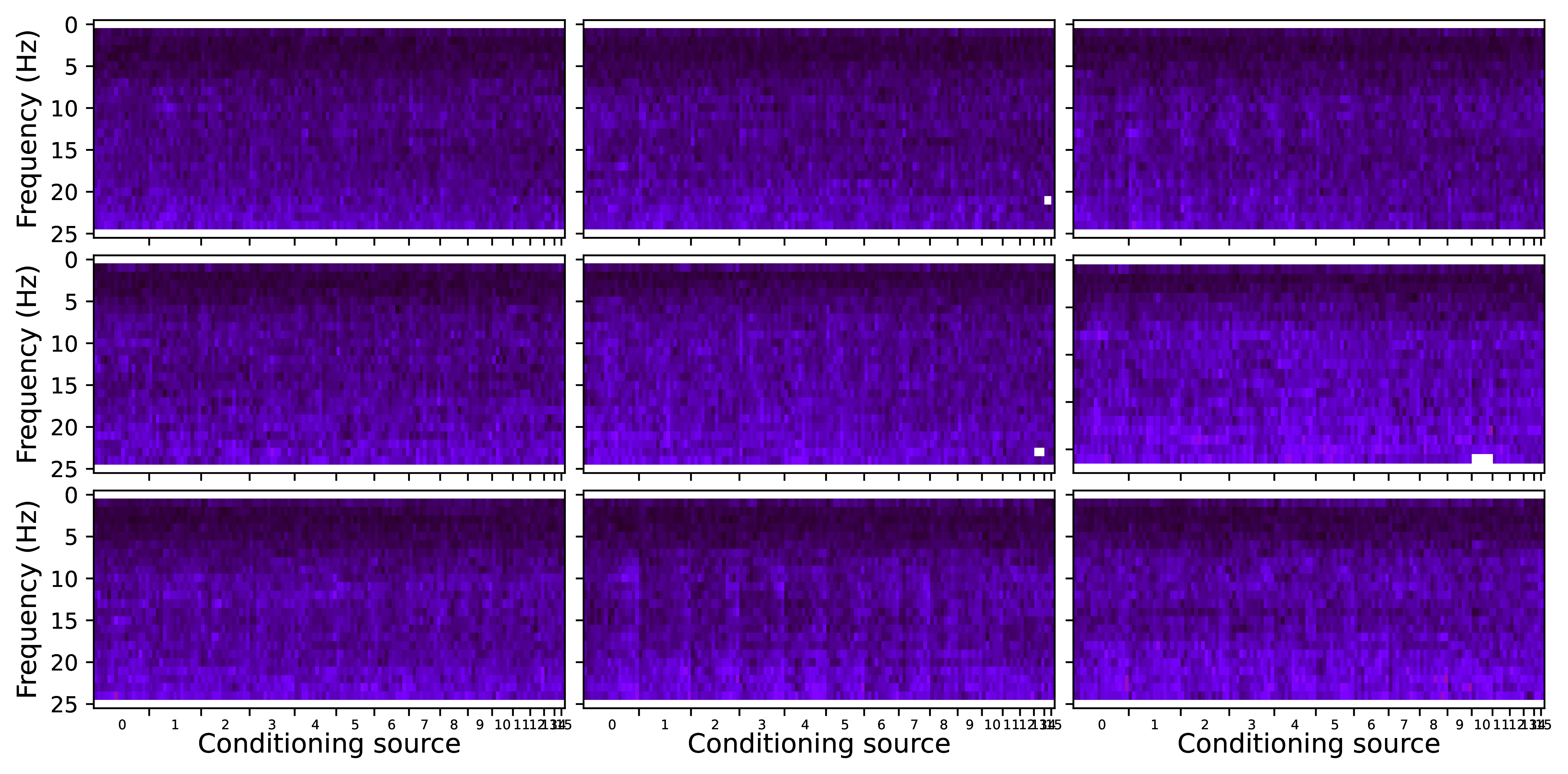

Supplement: S6 Fig — p-values of the energy test measuring the difference between the conditional distribution of coefficients at one source given those at another (the ‘conditioning source’), and a bivariate Gaussian with the same mean and covariance, for each frequency. Each column corresponds to the comparison for one pair of sources. Colours as in S2 Fig, white indicates p < 0.001. (TIF) [file pone.0297754.s007.tif]

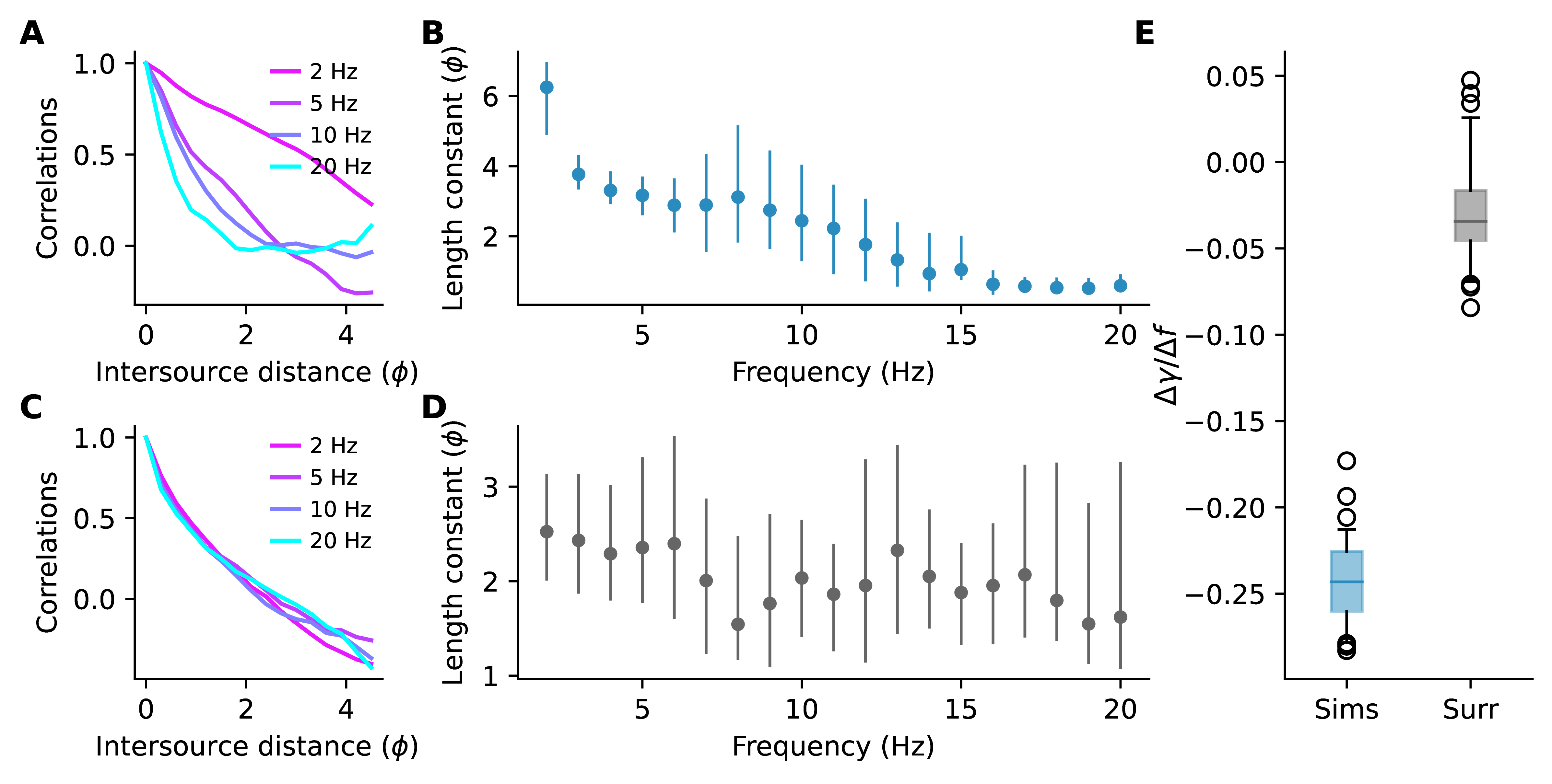

Supplement: S7 Fig — Compare to Fig 9. (TIF) [file pone.0297754.s008.tif]

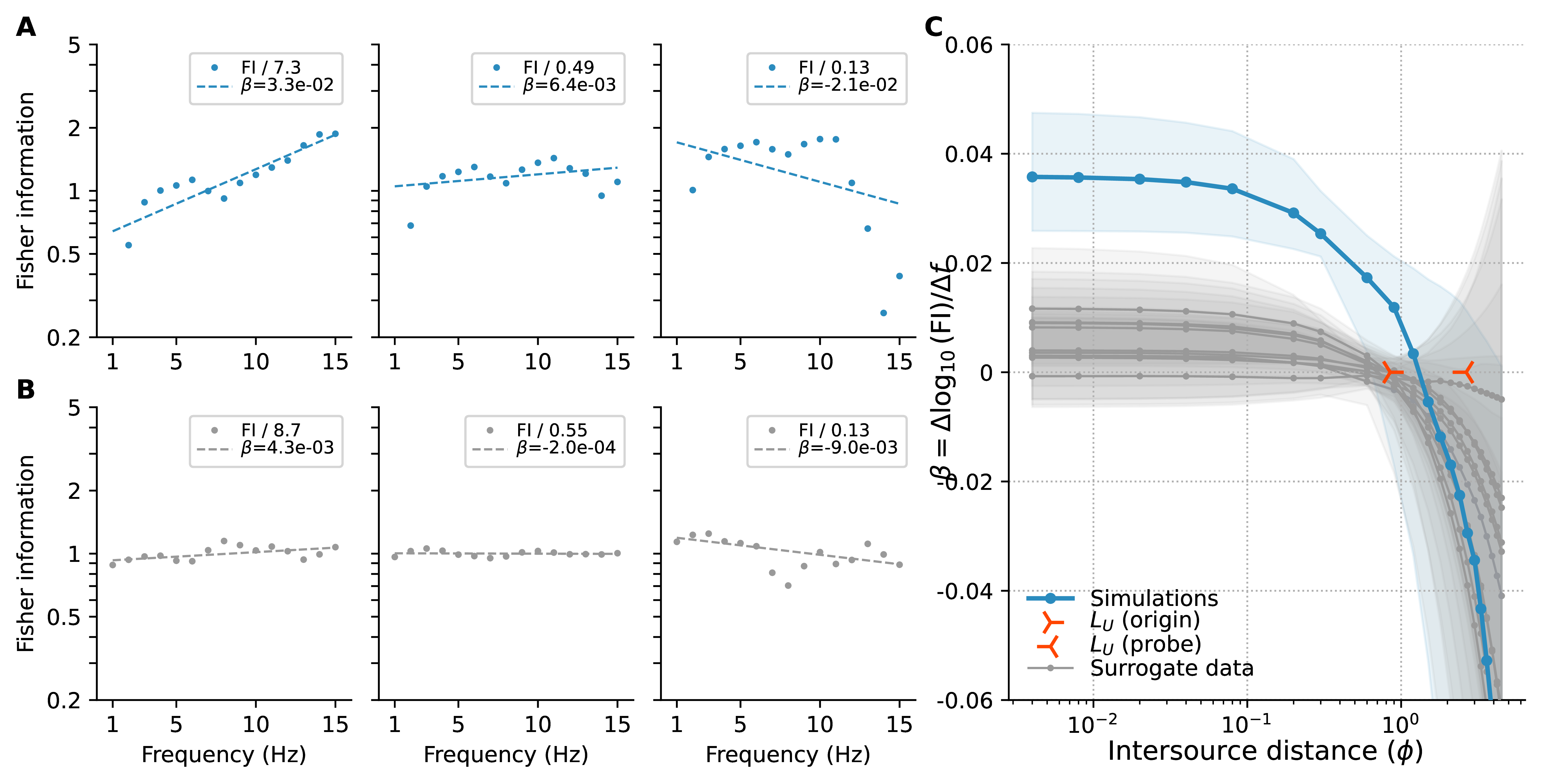

Supplement: S8 Fig — Compare to Fig 12. (TIF) [file pone.0297754.s009.tif]

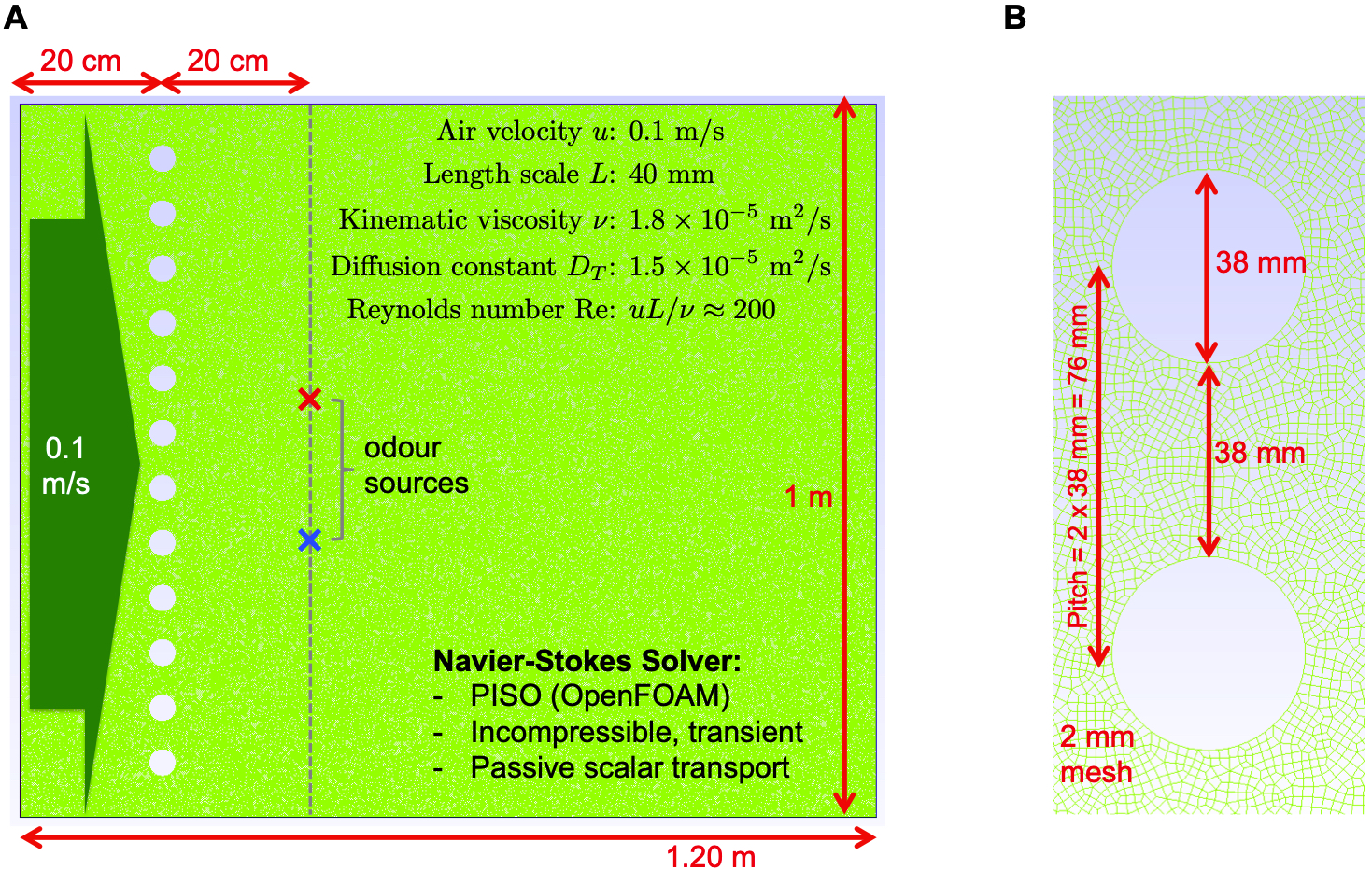

Supplement: S9 Fig — (A) Domain schematic and simulation parameters. Flow was from left-to-right. Vorticity was introduced into the flow by twelve cylindrical obstacles, vertically spaced evenly at a horizontal distance of 20 cm from the inlet (see also panel B). Point odour sources were placed at various vertical locations on the dashed line 40 cm horizontally from the flow inlet. Flow over these sources carried the odours to downstream probes. (B) Details of the obstacles and the simulation mesh. The cylindrical obstacles were 38 mm in diameter and evenly spaced 38 mm apart. The resulting center-to-center distances of 76 mm defined the pitch. The domain was discretized using a mesh with 2 mm resolution. (TIF) [file pone.0297754.s010.tif]

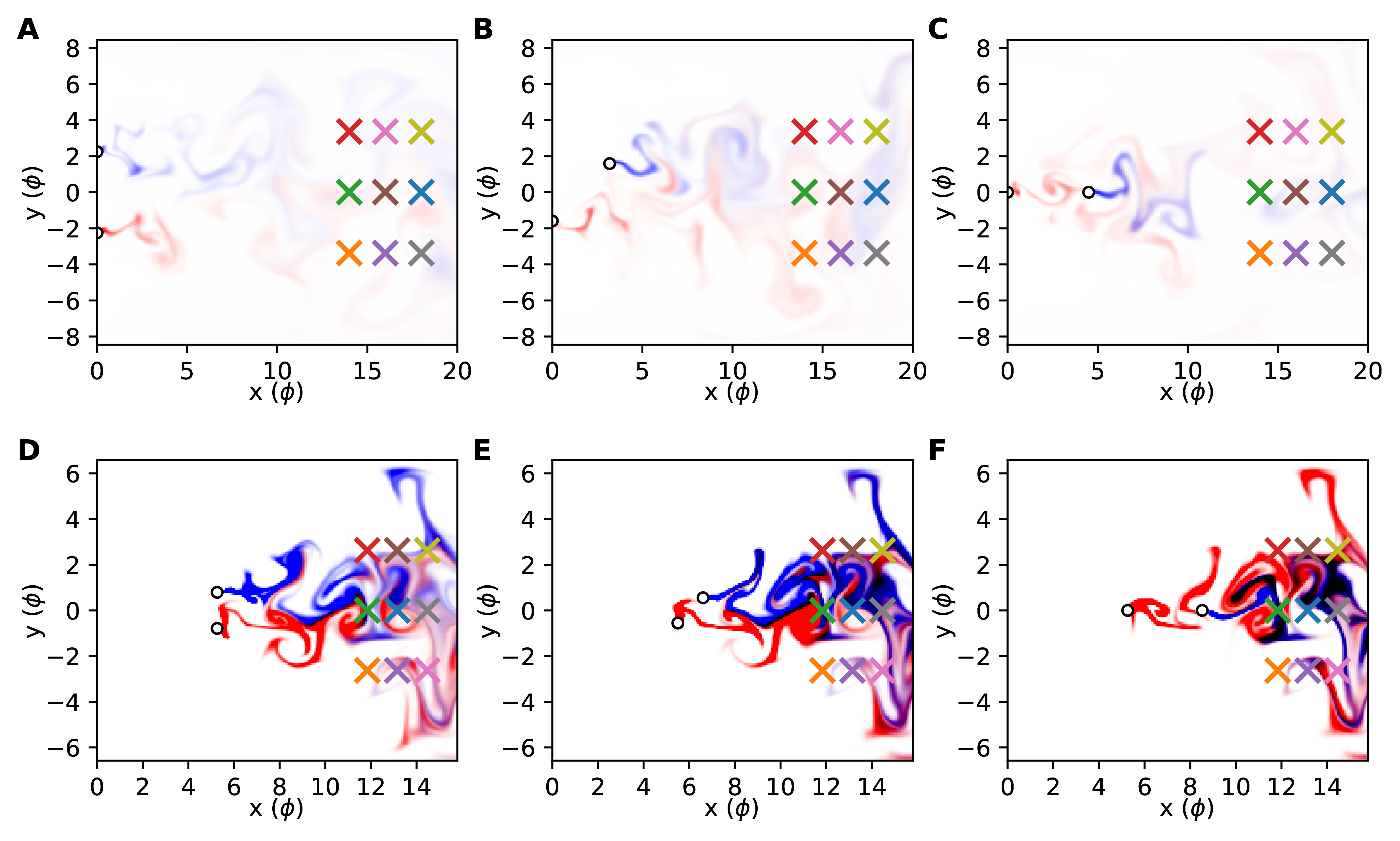

Supplement: S10 Fig — The full set of simulations and probe locations used in our study. Each panel shows a snapshot of the plumes from the most distal sources, with the two source locations indicated by the white ‘o’s. All simulations used 16 equally spaced sources, inclusive, between the two distal locations indicated, except for the simulations in panel F, which used 32 sources. The nine probe locations in each simulation are marked with ‘×’. (A) The main simulations used in our study, with sources transverse to the direction of the flow. The principal probe location that we discuss in the Main Text is at the blue ‘×’. (B) Simulations with sources at 45 degrees to the flow. (C) Simulations with sources parallel to the flow. (D) The principal supplementary simulations we analyze in the main text, with sources transverse to the flow. The principal probe location that we analyze is at the blue ‘×’. (E) Supplementary simulations with sources at 45 degrees to the flow. (F) Supplementary simulations with sources parallel to the flow. This set of simulations used 32 sources, rather than 16. (TIF) [file pone.0297754.s011.tif]

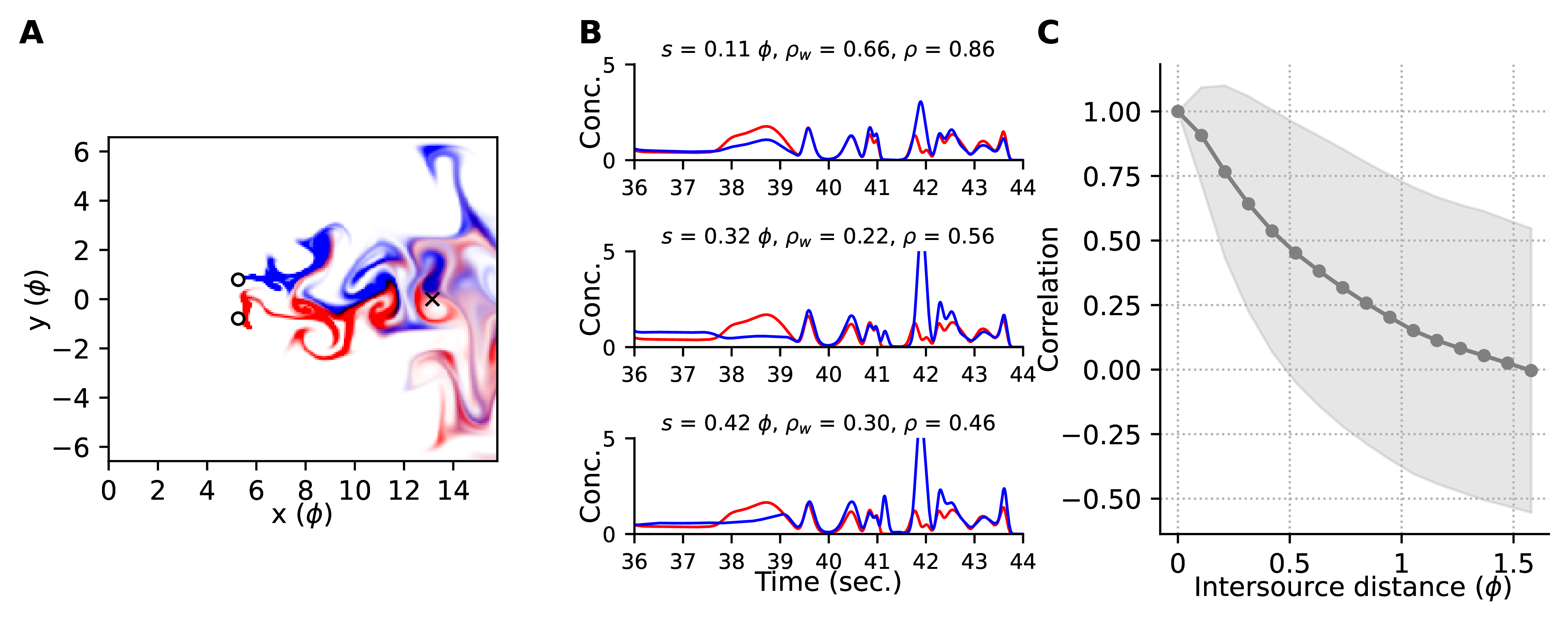

Supplement: S11 Fig — Compare to Fig 3. (TIF) [file pone.0297754.s012.tif]

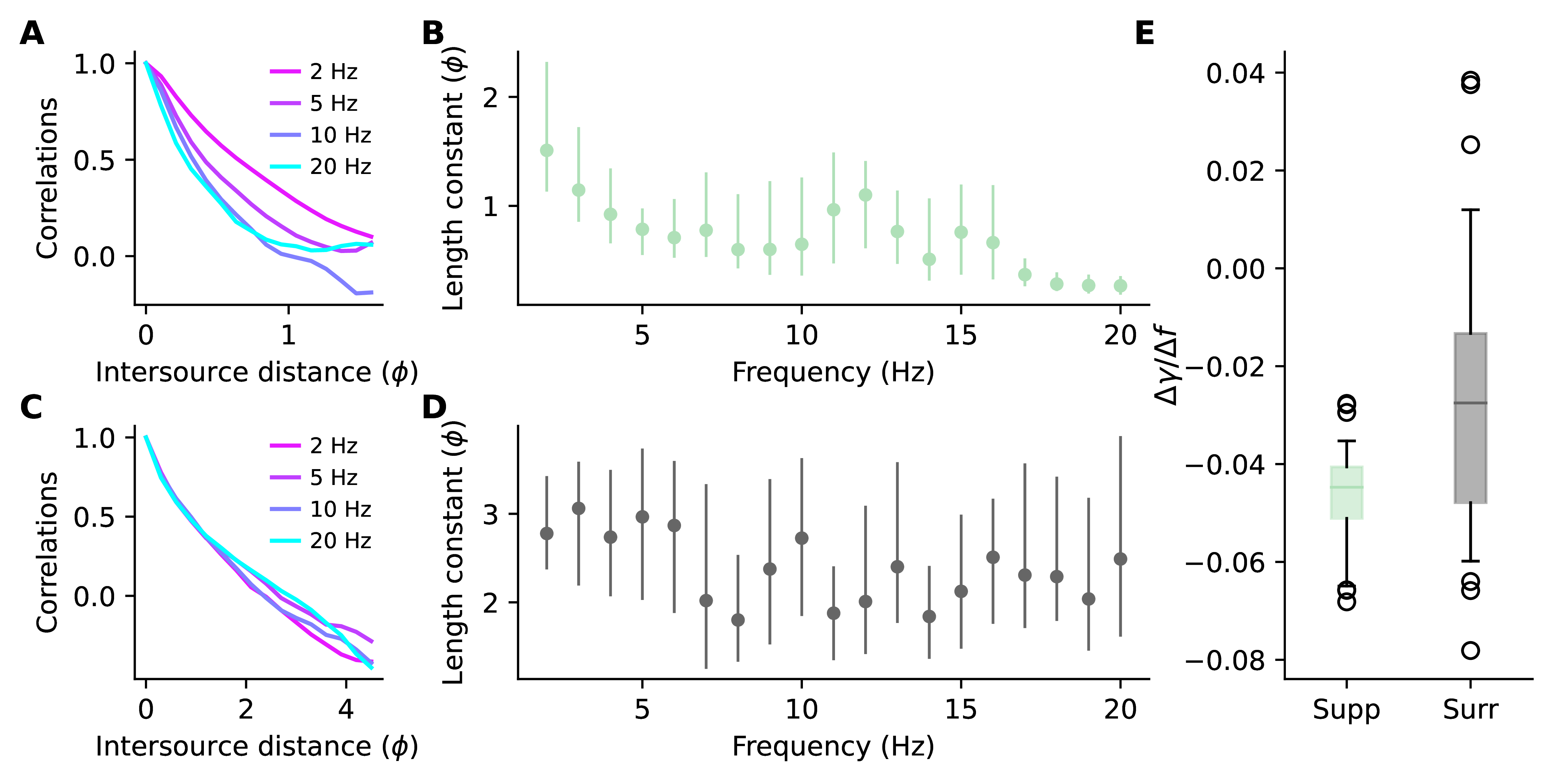

Supplement: S12 Fig — Compare to Fig 9. (TIF) [file pone.0297754.s013.tif]

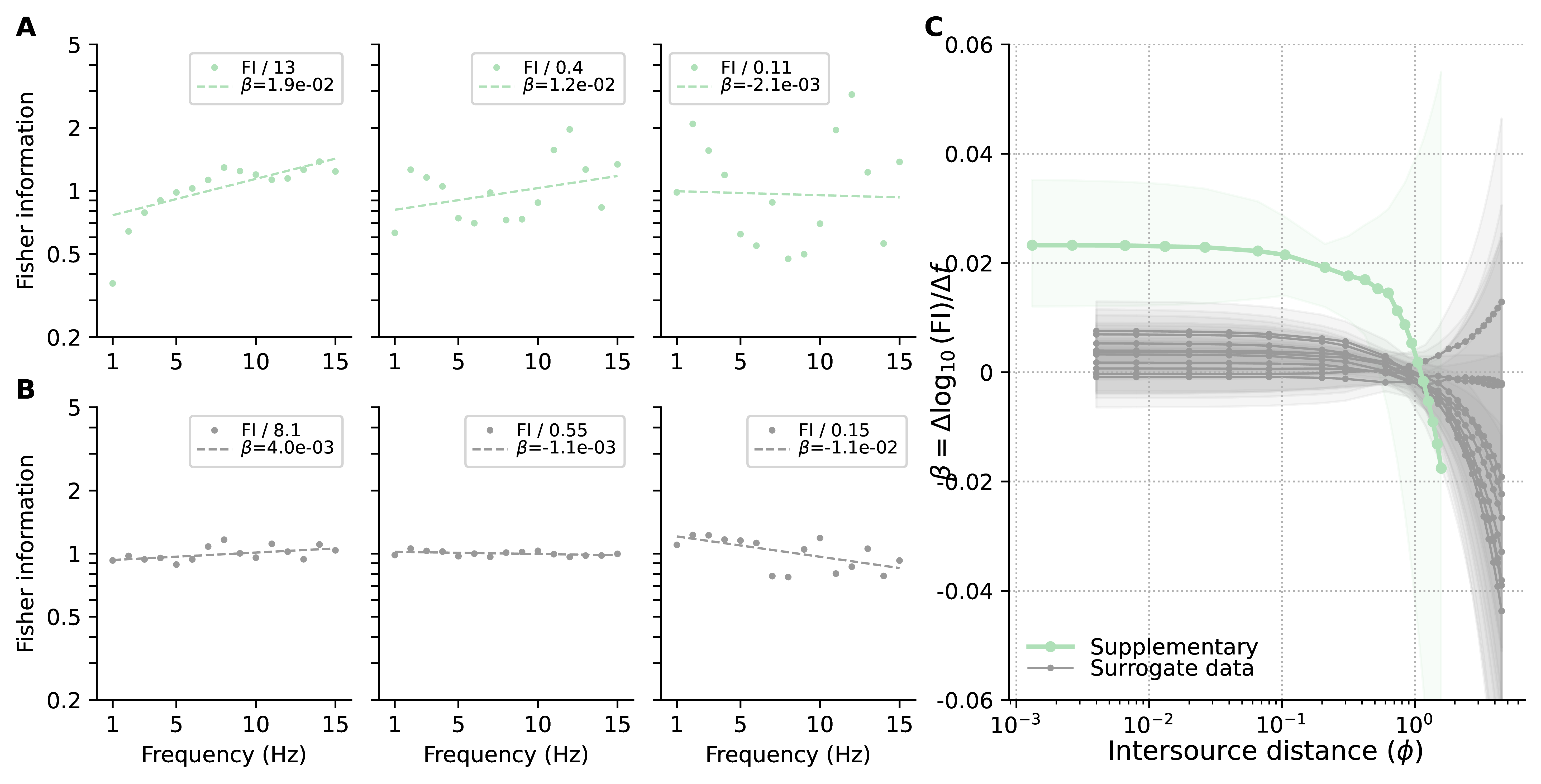

Supplement: S13 Fig — Compare to Fig 12. The fits are at for intersource distances of 0.1, 0.4 and 0.7 ϕ. The intersource distances for the supplementary data start at lower pitch values than for the data in the Main Text because the pitch for the supplementary simulations is ∼3× larger. (TIF) [file pone.0297754.s014.tif]

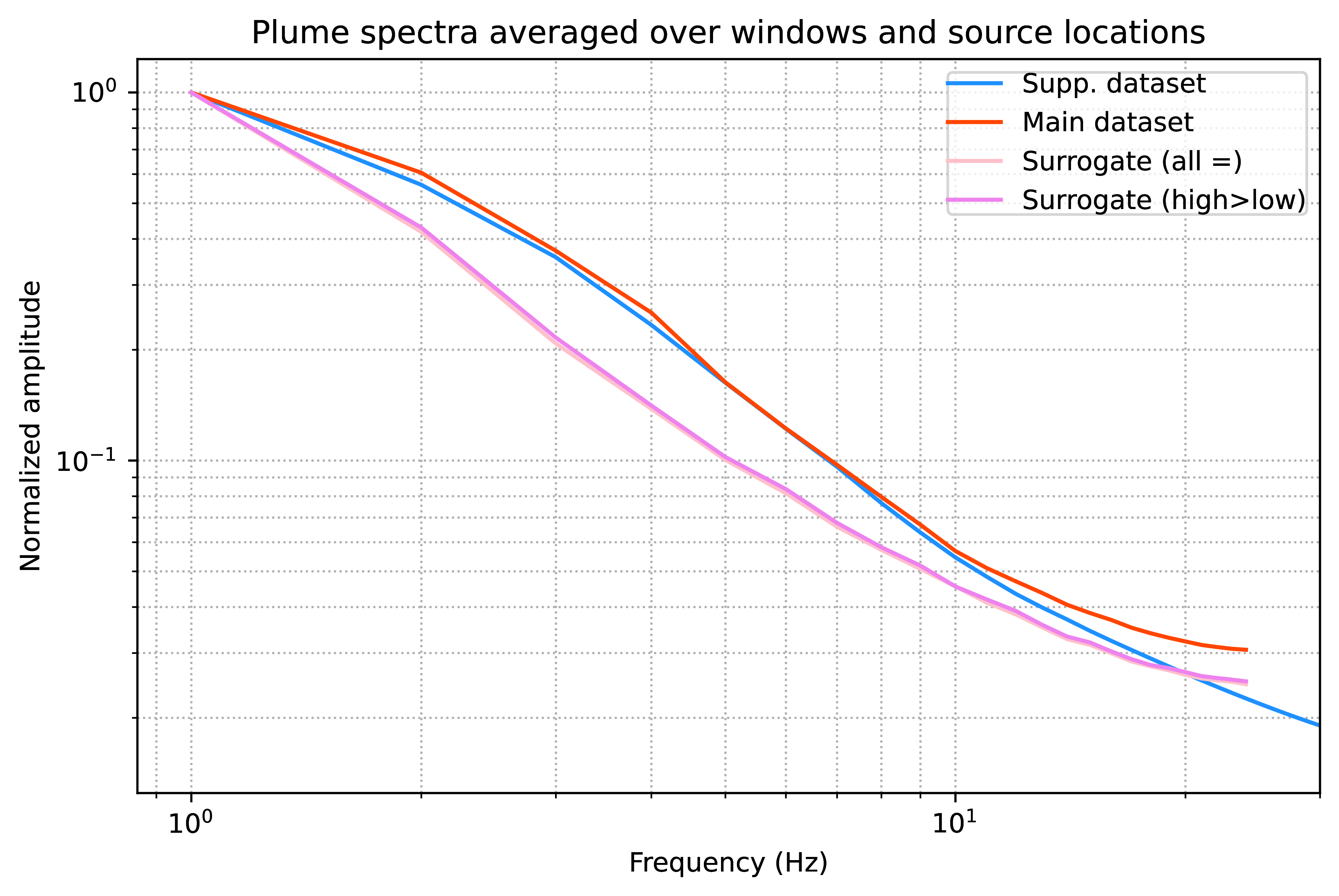

Supplement: S14 Fig — Discrete Fourier transforms were computed for consecutive 1-second windows that overlapped by 500 msec, amplitudes were averaged and scaled to have the same value at 1 Hz. Surrogate datasets are indexed by their information content (‘all=’: all frequencies equally informative; ‘high>low’: high frequencies more informative than low frequencies). The surrogate datasets (all =) and (high > low) were used in Fig 11 panels B, and C, respectively. (TIF) [file pone.0297754.s015.tif]

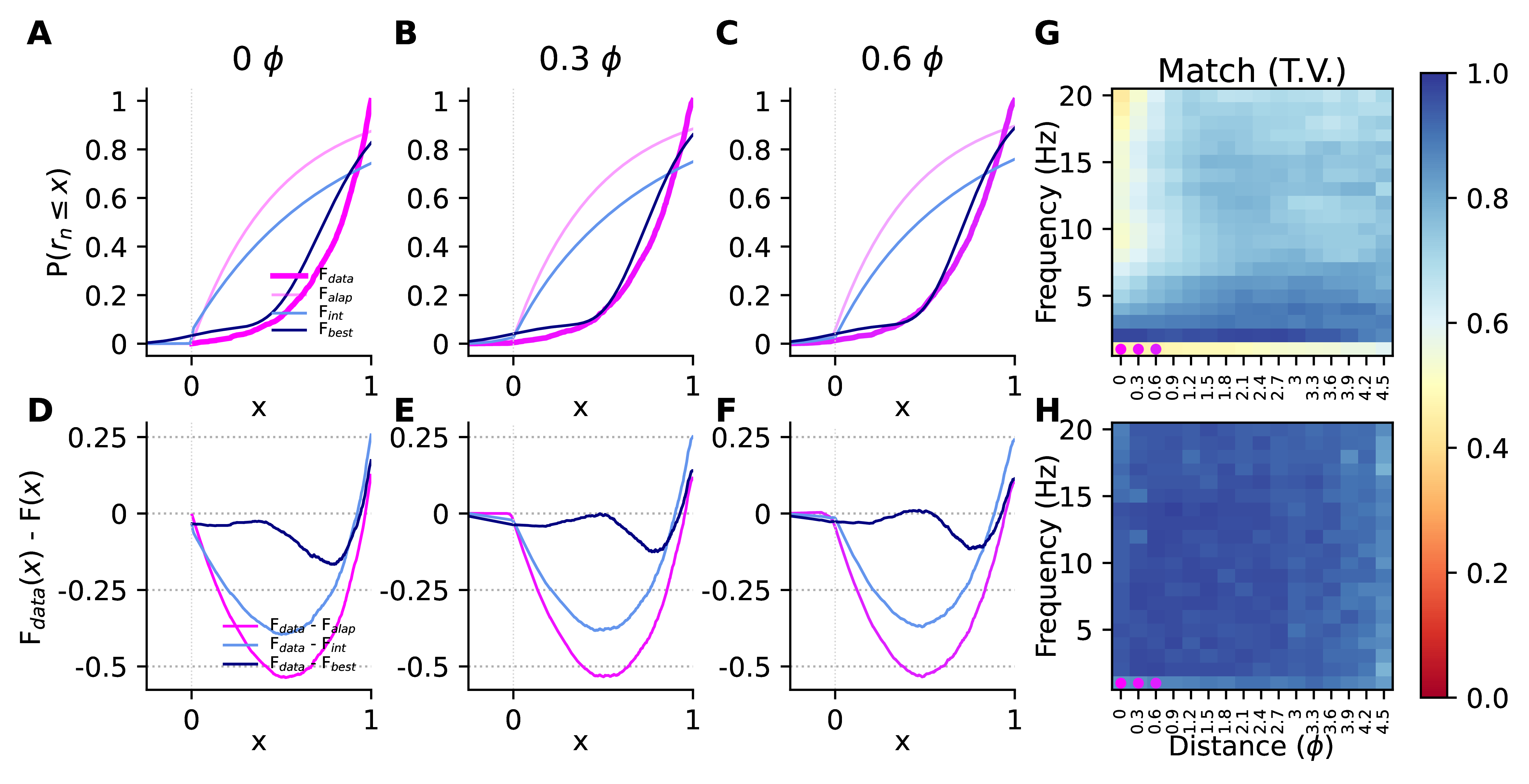

Supplement: S15 Fig — As in Fig 7 but showing the fits to the 1 Hz data, highlighting the poor fits to the data. (TIF) [file pone.0297754.s016.tif]

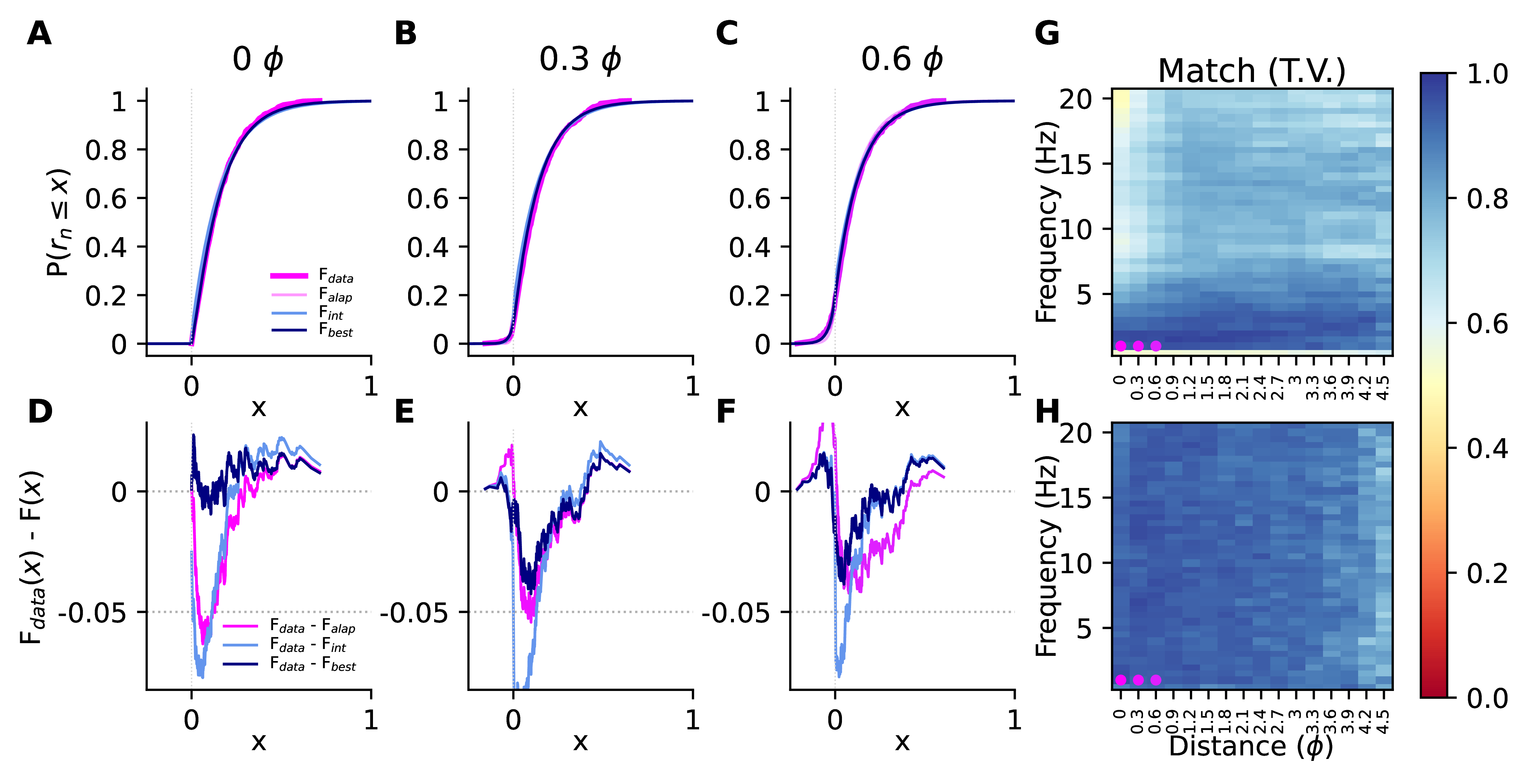

Supplement: S16 Fig — As in Fig 7 but showing the fits to the 1 Hz data, and when computing all statistics over 2-second Hann windows instead of the 1-second windows used in Fig 7. (TIF) [file pone.0297754.s017.tif]

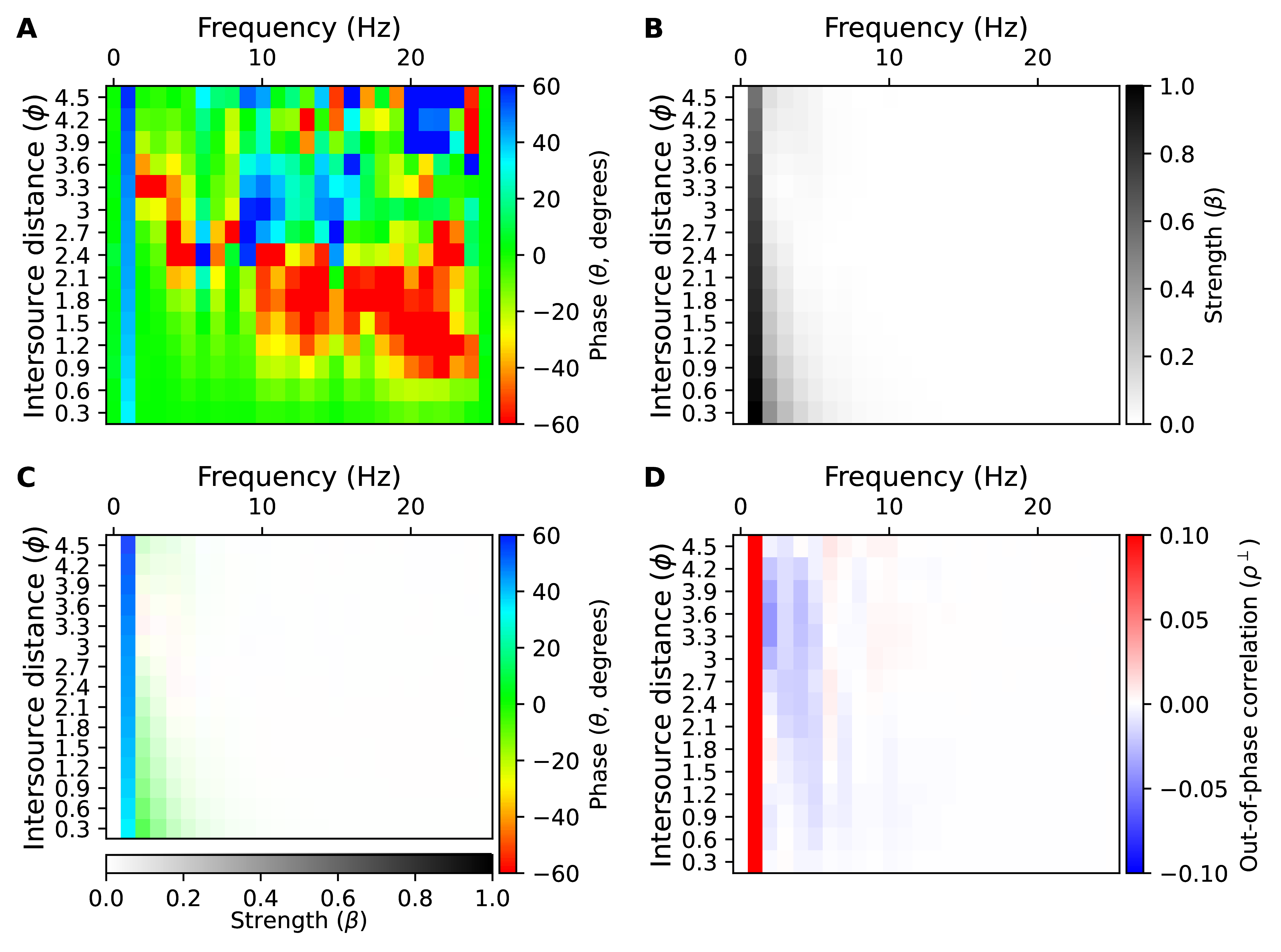

Supplement: S17 Fig — Coupling of concentration profiles from two sources at each frequency as a function of intersource separation, expressed in terms of (A) phase and (B) strength of the coupling. (C) Strength (saturation) and phase (hue) together. (D) Out-of-phase correlations, computed as β sin(θ). (TIF) [file pone.0297754.s018.tif]

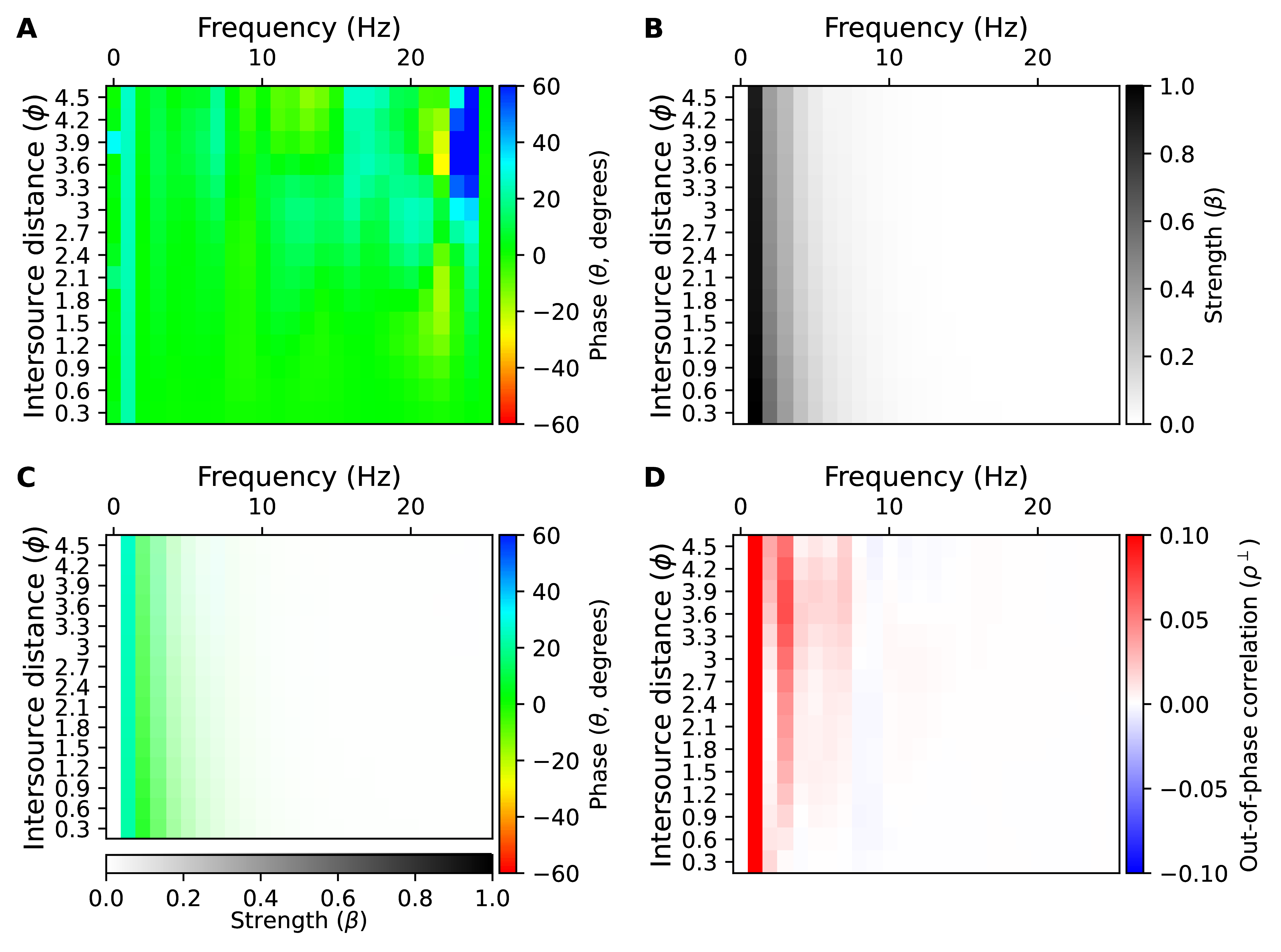

Supplement: S18 Fig — (TIF) [file pone.0297754.s019.tif]

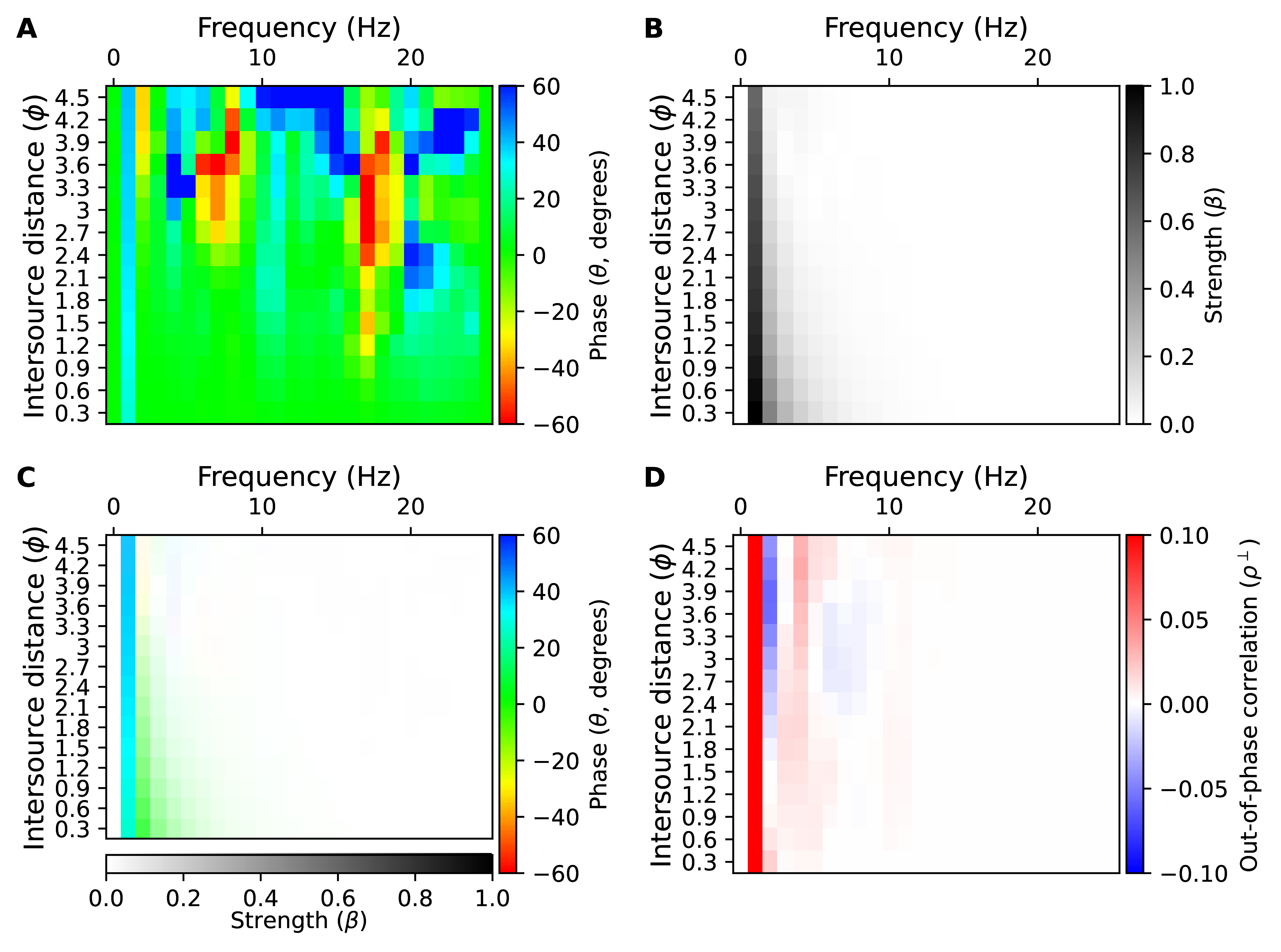

Supplement: S19 Fig — (TIF) [file pone.0297754.s020.tif]

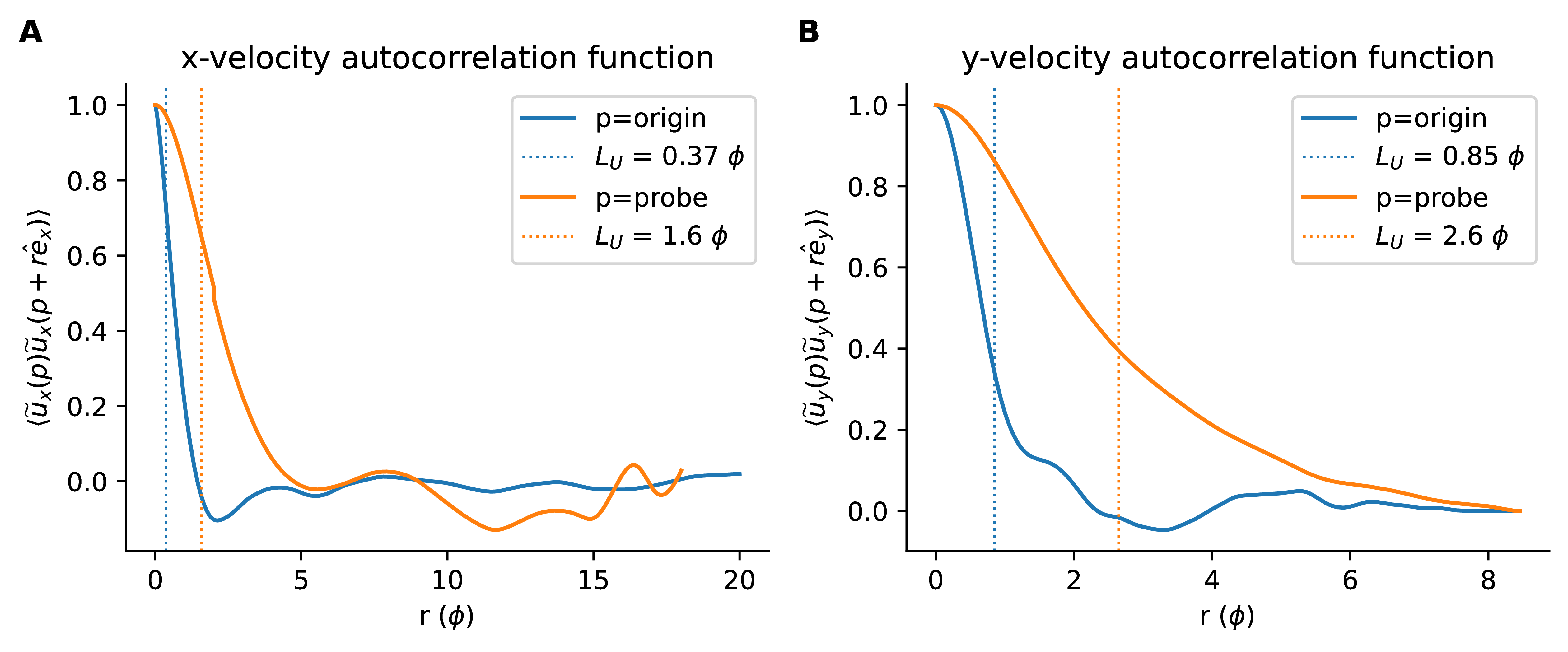

Supplement: S20 Fig — (A) Velocity in the x-direction (parallel to the mean flow) autocorrelated along the same direction. There are fewer data points at the ‘probe’ location since the largest x-displacement at that location is to the origin, while the largest x-displacement for the ‘origin’ extends past the probe location. (B) Velocity in the y-direction (perpendicular to the mean flow), autocorrelated along the same direction. The maximum displacement is approximately half that of panel A because the data in that panel spans the entire width of the simulation domain, while the data in this panel only extends from the midline to the upper and lower boundaries. (TIF) [file pone.0297754.s021.tif]

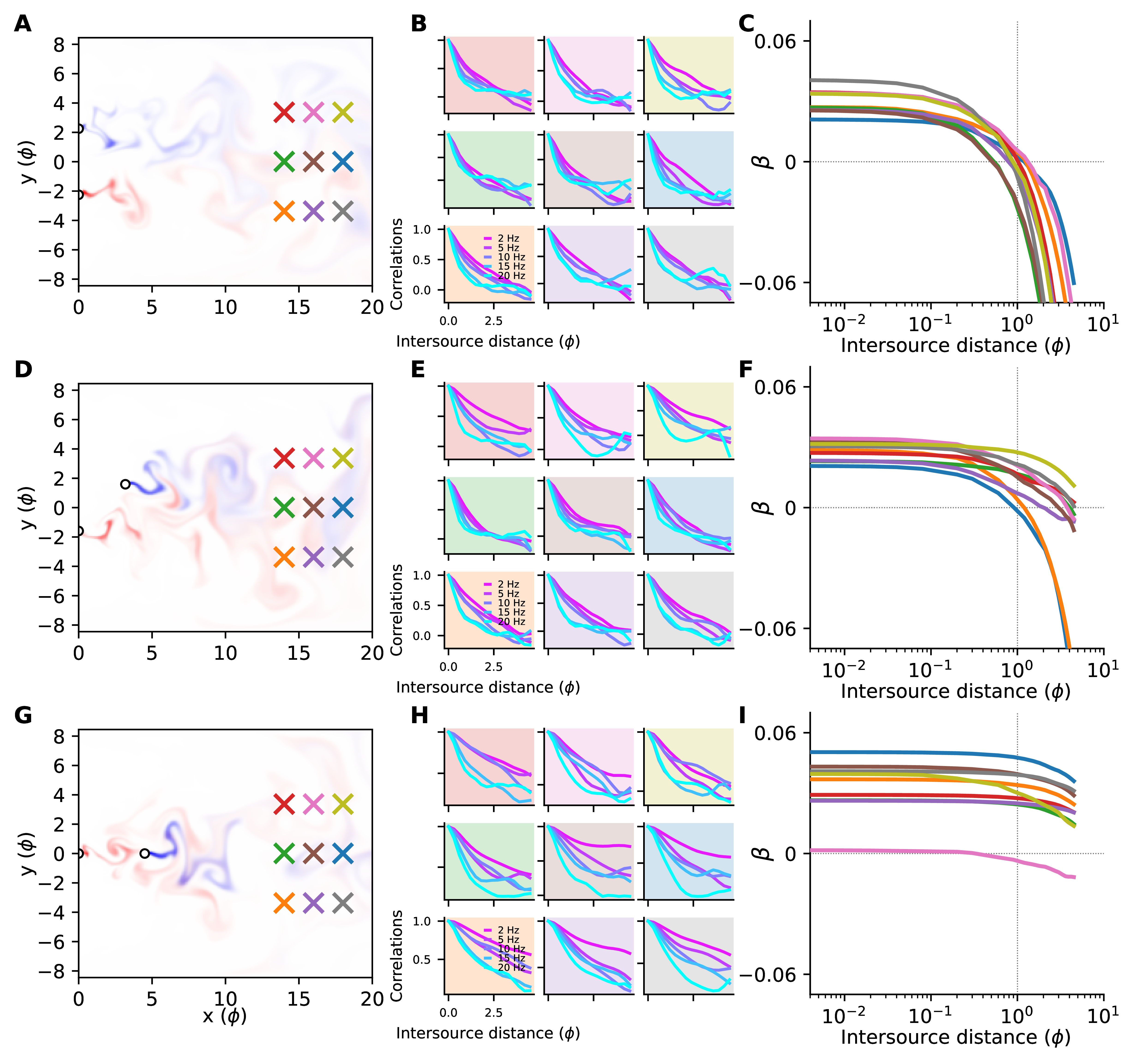

Supplement: S21 Fig — As in Fig 13 but using a 2-second Hann window. (TIF) [file pone.0297754.s022.tif]

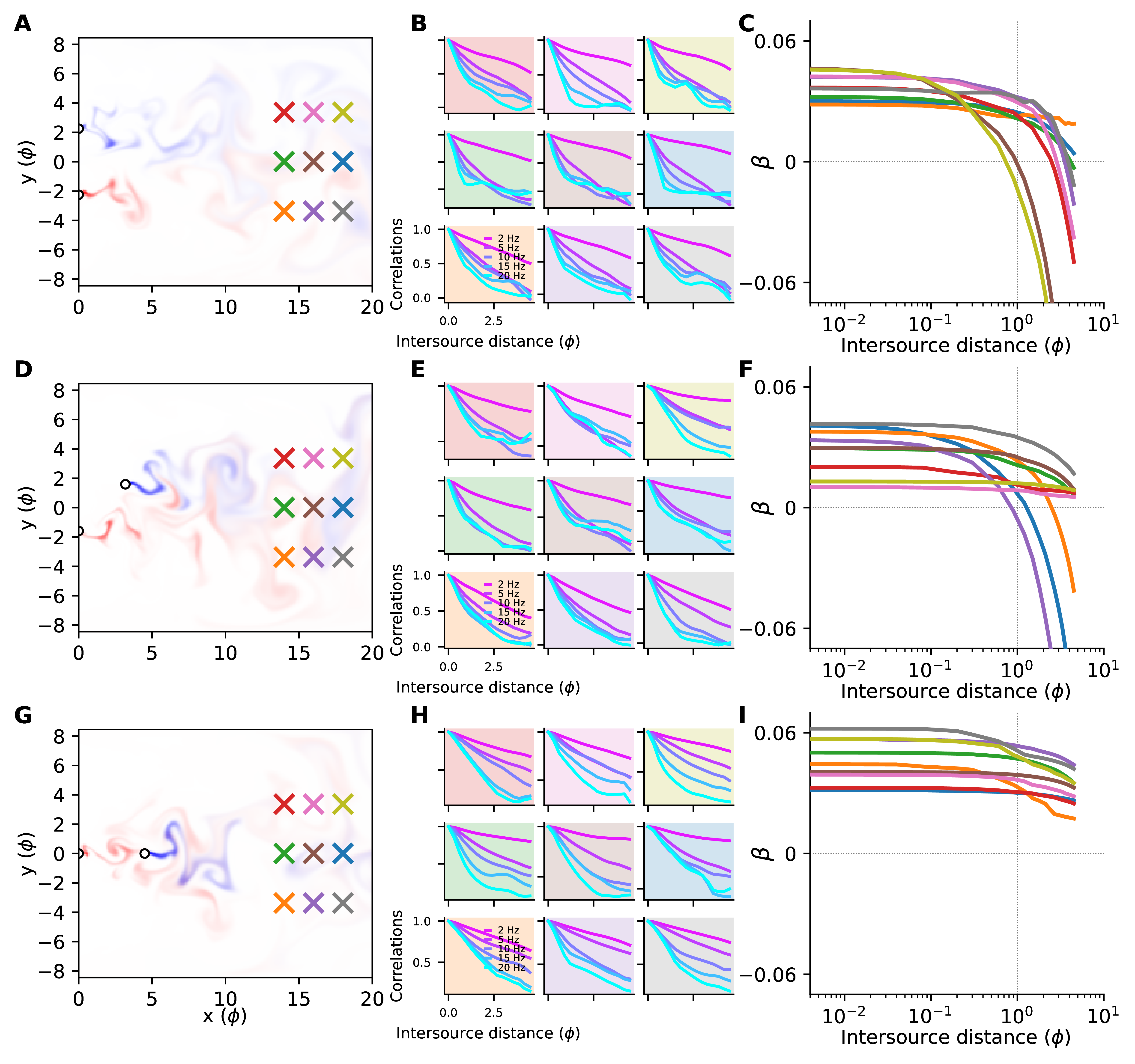

Supplement: S22 Fig — As in Fig 13 but using a 0.5-second Hann window. (TIF) [file pone.0297754.s023.tif]

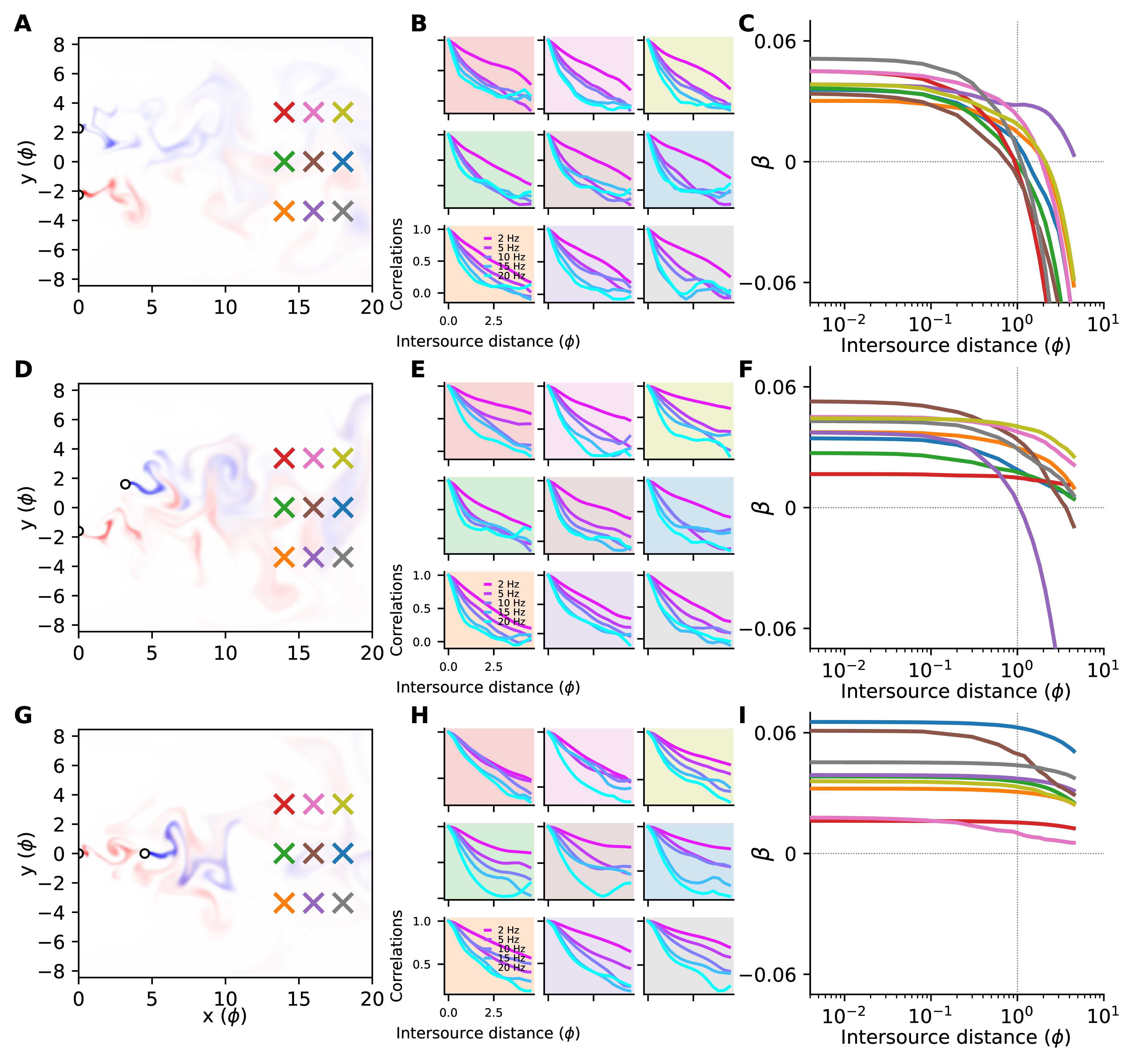

Supplement: S23 Fig — As in Fig 13 but using a 1-second Kaiser-16 window. (TIF) [file pone.0297754.s024.tif]

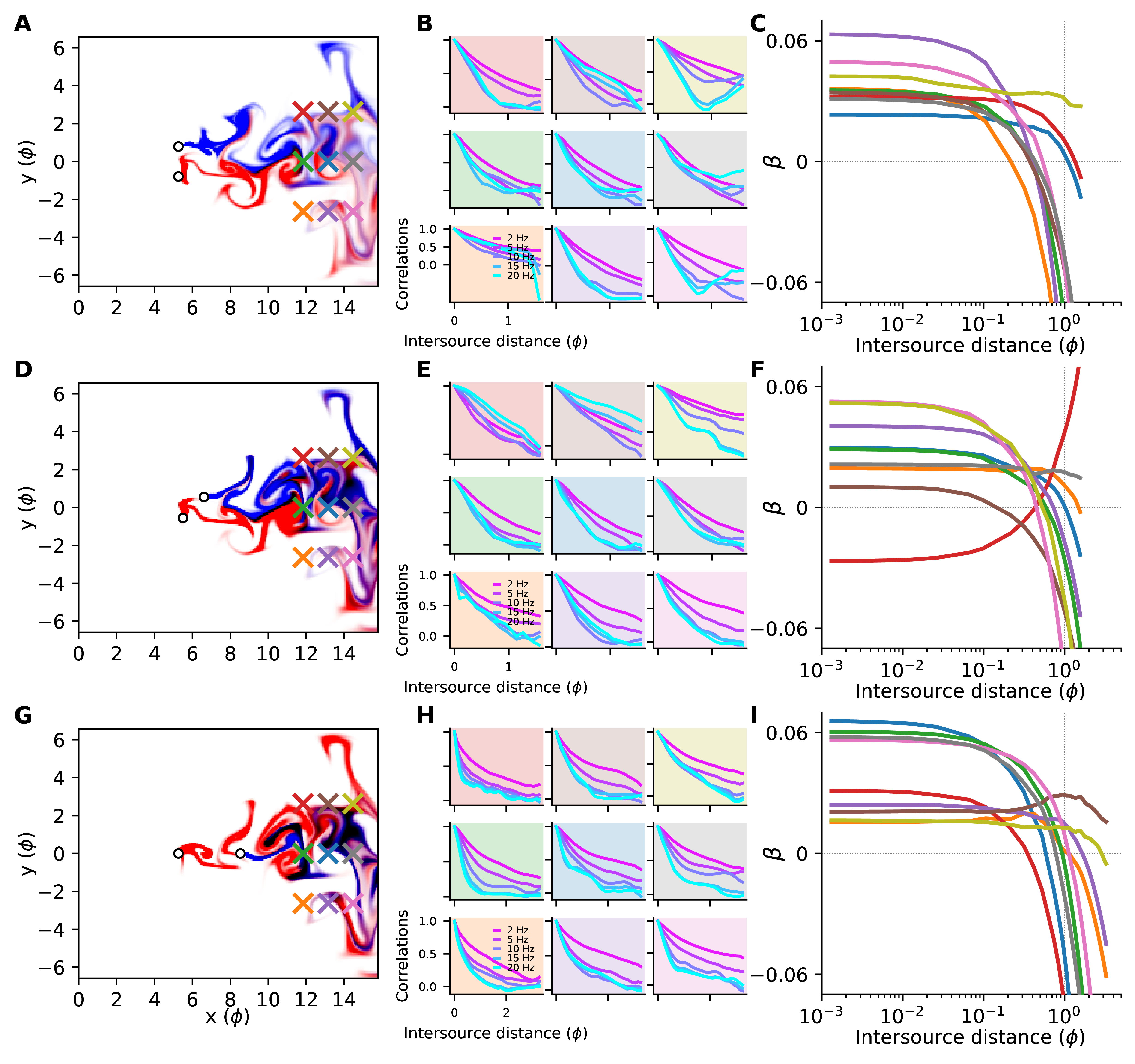

Supplement: S24 Fig — As in Fig 13 but for the Supplementary simulations. (TIF) [file pone.0297754.s025.tif]

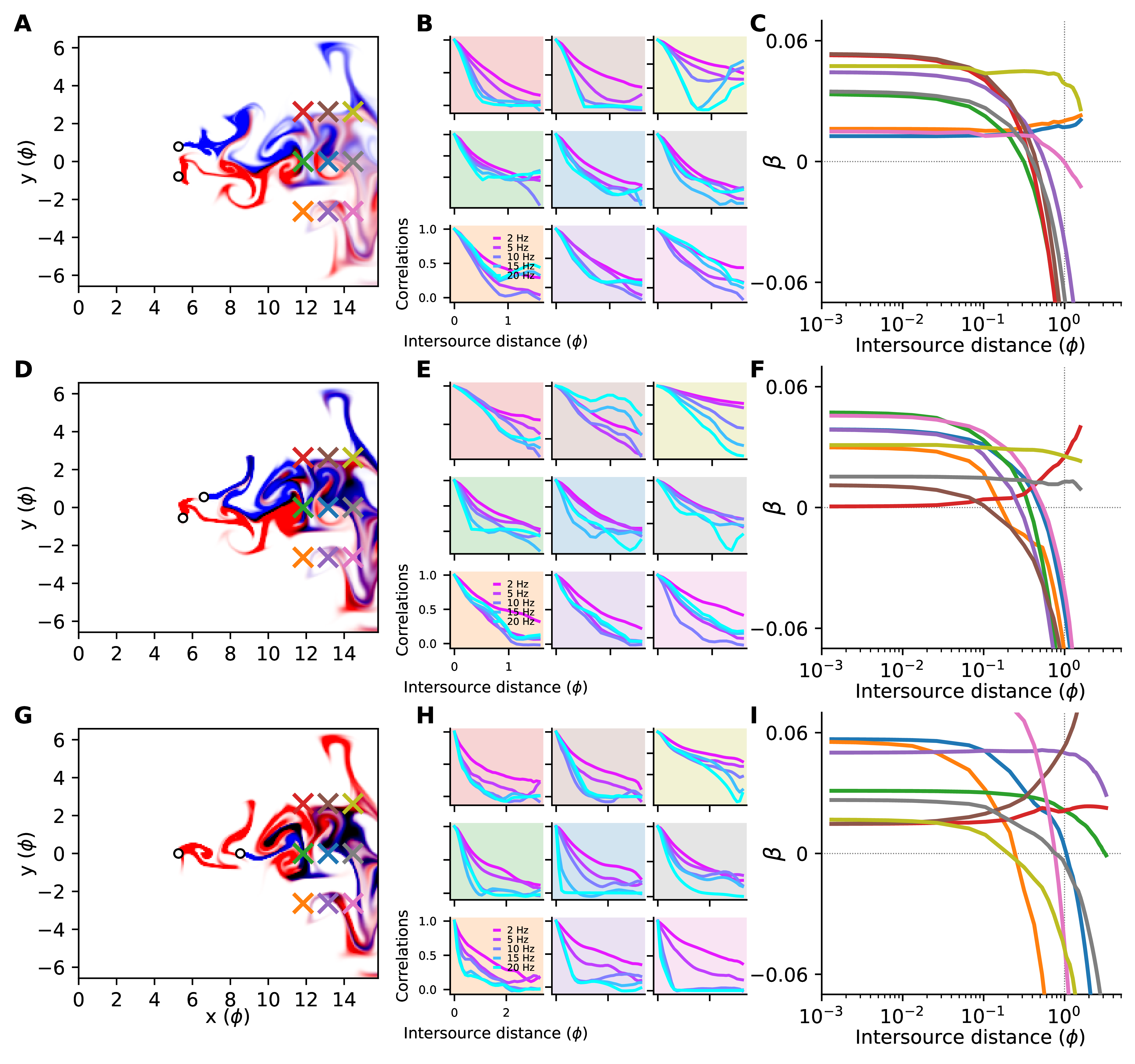

Supplement: S25 Fig — As in S24 Fig but using a 2-second Hann window. (TIF) [file pone.0297754.s026.tif]

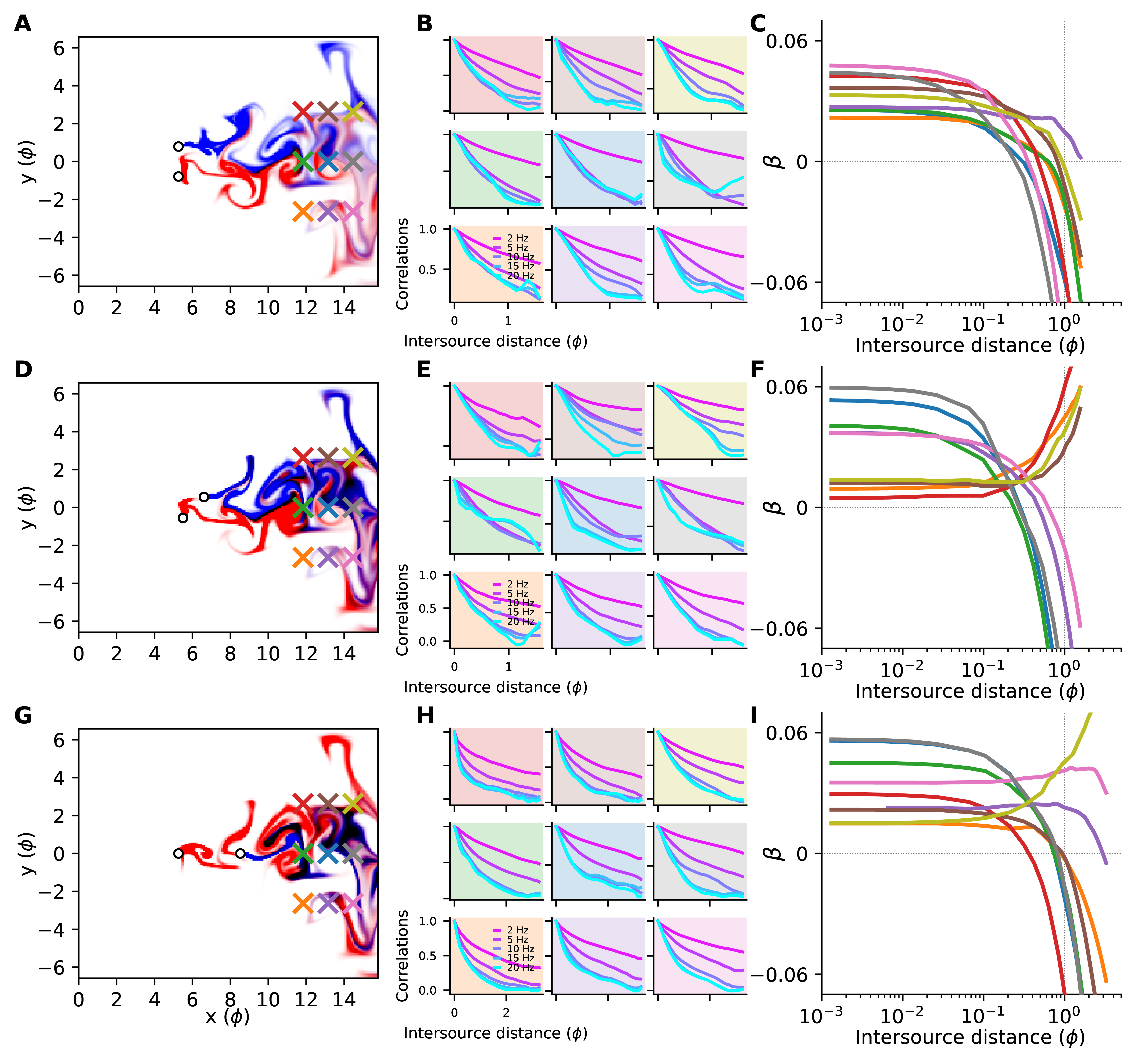

Supplement: S26 Fig — As in S24 Fig but using a 0.5-second Hann window. (TIF) [file pone.0297754.s027.tif]

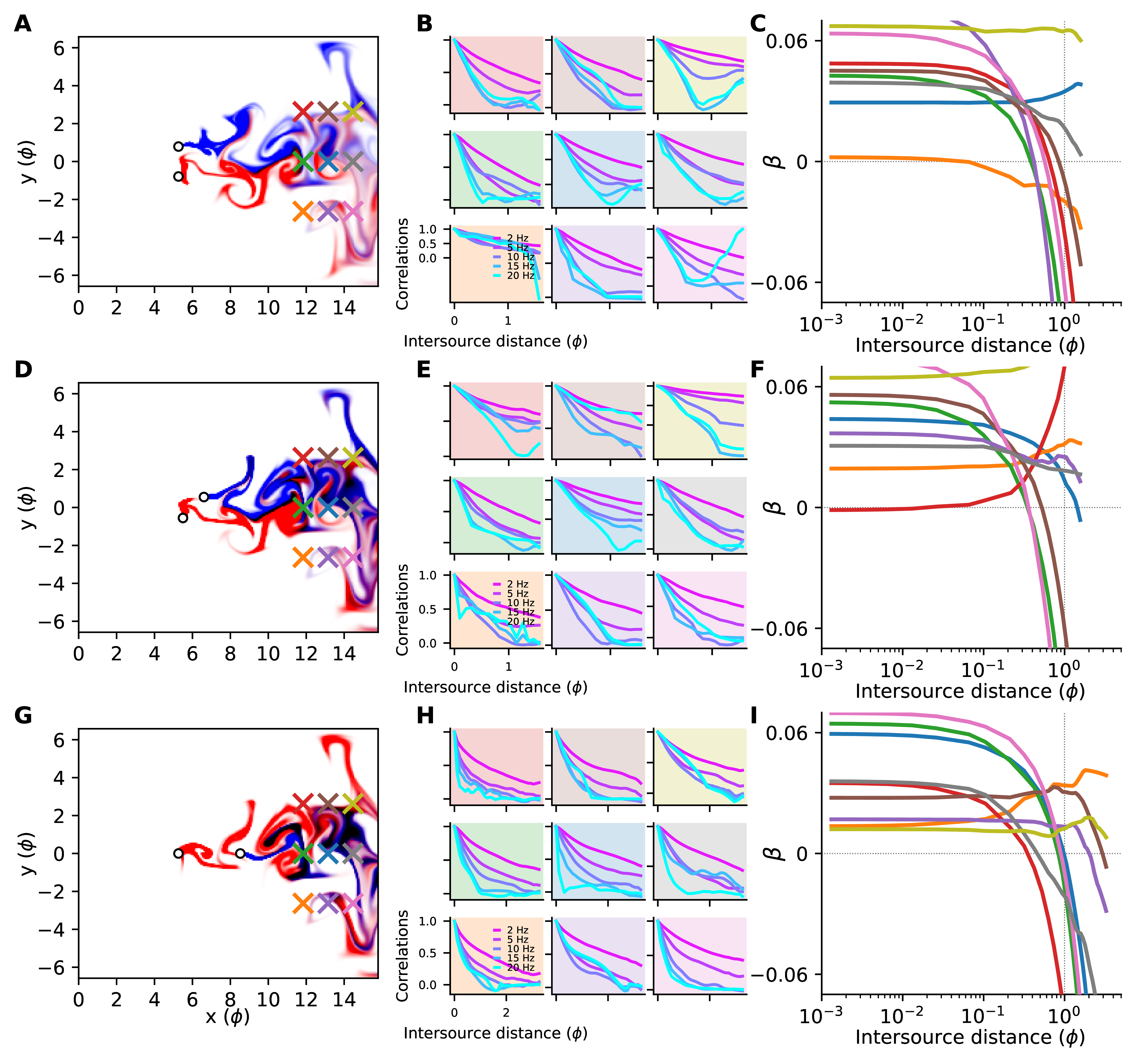

Supplement: S27 Fig — As in S24 Fig but using a 1-second Kaiser-16 window. (TIF) [file pone.0297754.s028.tif]
