# Supplementary material for: Quantifying spectral information about source separation in multisource odour plumes
Source: PLoS One. 2025 Jan 10;20(1):e0297754. doi: 10.1371/journal.pone.0297754 (PMC11723556; doi:10.1371/journal.pone.0297754)
Supplement: Attachment — Submitted filename: point-by-point-rebuttal.pdf [file pone.0297754.s029.pdf]

Reviewer #1:

Major points

1) Firstly, I am a bit worried about the generality of their statements. Their analysis appears to be focused on one quite specific setup in which:

- The sources are separated only in the direction orthogonal to the mean wind. What happens if they are instead shifted in the direction parallel to it (i.e. one behind the other)? Does their main argument still hold (high frequencies being more informative as sources are closer)? This is not obvious to me. Then, depending on the outcome of such analysis, I would recommend to at least comment in the discussion on the general case in which the displacement vector between the two sources forms an angle $0<\theta<\pi/2$.
- The correlations are measured only in one point in space. How does the Fisher information about the intersource separation change with the distance from the sources? Is there any qualitative change in the colormaps in Fig. 11 when shifting the point in either the crosswind or downwind direction?

We thank the reviewer for these two important points regarding the generalizability of our results. Similar sentiments were expressed by Reviewer 2. The bulk of our efforts in the revision were towards addressing these points. First, we analyzed the plumes at 8 additional locations in addition to the one presented in the submitted manuscript. These locations were displaced from the original location either in the crosswind direction, the downwind direction, or both. This yielded 9 probe locations for our original set of simulations of crosswind sources. We then performed two additional simulations in which the sources were placed either oblique to the flow, or parallel to the flow, just as the reviewer recommends. Finally, we repeated this entire procedure for the Supplementary simulations, which used a slightly different domain geometry and different simulation framework than those in the Main Text.(We will refer to these as the Main and Supplementary simulations). We thus increased the number of probe locations from 2 (one location in one plume in the Main simulations, and one location in one plume in the Supplementary simulations), to 54 (9 locations x 3 plumes in the Main simulations, and the same number in the Supplementary simulations). All of the source geometries and probe locations we tested are shown in Figure S10.

The new Figure 13 summarizes the results of the analysis at the 27 probe locations in the Main simulations. Panel A shows a snapshot of the crosswind source configuration and the locations of the 9 probes within it - the blue probe is the one originally analyzed. Panel B shows the decay of correlations with intersource distance at several example frequencies for each probe location. The blue subpanel is for the original probe location. Comparing that subpanel to the others shows that the same qualitative effect holds in each probe location, with high frequency correlations decaying faster than low frequency correlations when sources are close together. Panel C shows the 'elbow' plots indicating whether high or low frequencies are more informative at each intersource distance - the blue curve is for the probe location originally analyzed. All of the other curves show the same qualitative trend, with high frequencies being more informative when sources are closer together.

Panels (D-F) similarly summarize the results for the oblique source configuration, and panels (G-I) do so for the streamwise source configuration. All of these data show the same qualitative effects as what we observed in the original probe location and source configuration - that high frequency correlations decay faster than low frequency correlations, which leads to high frequencies being more informative than low frequencies when sources are close together.

We saw qualitatively similar effects in our Supplementary simulations (Fig S24), except that in those simulations, two probe locations in the oblique configuration (red and brown data in Panels D, E and F) some high frequencies decayed slower than low frequencies. This was reflected in the red probe location as low frequencies being more informative than high frequencies for sources close together (red curve in panel F). In the Discussion we speculate that this could be due to those probe locations being on the edge of the plume, and being closer to the sources than in the Main simulations.

In summary, we saw qualitatively the same effect as we saw at the original probe location in the plume created by the crosswind sources for all other probe locations and source configurations in the Main simulations, and at most probe locations and source configurations in the Supplementary simulations.

2) The time window used to compute the correlations seems crucial for any conclusions to be drawn. A natural question would then be how this effective time window of "observation" is reflected in biological systems. Is there a typical one that can be considered for animals/insects? If so, how does this compare with the values used in their numerical simulations? The authors quickly mention this connection at lines 375-381 in the discussion, but I would at the very least ask them to comment further on this point by addressing more precisely my question and citing the relevant literature.

The reviewer raises an important question to which we can only provide a partial answer. The integration time of single neurons is often stated to be 10 - 100 ms, based on the electrical time constants of cell membranes. Networks of neurons with appropriate connectivity can have much larger integration times (e.g. Ganguli, Huh, Sompolinsky 2008). Mice make many decisions (e.g. about odor identity) within a few 100s of ms (e.g. Abraham et al 2004, Uchida & Mainen 2003, Rinberg et al 2006, Resulaj & Rinberg 2015), suggesting that observation times windows in mice are of this length or shorter. We chose to remain agnostic on what typical values for time windows are, and instead performed our analysis at two additional time windows, one twice as long and the other half as long as the 1-s window used in the text. These results are shown in Figures S21 and S22 for the Main simulations and S25 and S26 for the Supplementary simulations, and showed qualitatively the same effects as what we observed using 1-second window, including the additional variability for probes near the edge of the plume in the Supplementary simulations. Therefore we believe that our results are robust to window sizes at least within the 500ms - 2 second range.

Minor points:

1) While reading Sec. 2.2.1, I was confused by the fact that, on the one hand, the authors try to improve the model of correlations derived in the previous section. On the other hand, to the best of my understanding, they later on (Sec. 2.3) forget about this corrections and still

use the most simplified model to generate all the analytic results thereafter. At this point, if I am not mistaken, why not moving this section with the improved models in the Supplementary Material and include some of the calculations leading to the final expression of the Fisher information in the main body instead? I think this would improve the readabiity of this technical (and very important) part of the paper.

We thank the reviewer for this comment - they are correct that we develop improved models of correlations, but then use the simpler models when computing Fisher information. We believe that these models are an important contribution in their own right as they accurately capture the correlation distributions at most frequencies and intersource locations, highlight the importance of intermittency, show that the probability distributions used in the single-source concentration modeling literature readily apply to describing correlations, and can provide a useful substrate for more sophisticated computations of Fisher information. However, we also agree with the reviewer that showing their full derivation at such an important point in the text can make the text harder to read. We have therefore significantly shortened the Main text describing these more advanced correlation models, relegating the derivations to the Methods and Supplementary information. We hope these changes improve the readability of the text, while also allowing us to present the more accurate correlation models.

2) I would recommend the authors to make more precise citations in most of the sentences in the introduction and avoid putting references in bulks. Note, for e.g., how the first sentence does not reference any paper in the literature while bringing up important points. In that regard, I would also suggest to expand the introductory paragraph about olfactory search in complex or turbulent environments*. I am sure this would definitely strengthen the problem statement while increasing the visibility of this research.

We apologize for bulking references, and for the lack of references at the locations the reviewer indicates. Based on the reviewer's suggestions we have now rewritten the introductory paragraphs, made our citations more specific, and contextualized our work within the research on olfactory research guided by the references the reviewer provided.

3) Can the results presented here be useful as a basis to implement optimal navigation strategies in complex landscapes? I am thinking of active agents across all scales (from microswimmers to moths, mice, birds or even robots). In my opinion, it could be a nice potential application that can be inserted as a further outlook in the discussion section**.

We are grateful to the reviewer for pointing out the possible applications of our work to optimal navigation and thank them for the references to the relevant literature. We used these as a basis for our new Discussion section 3.3 where we describe how our findings may be employed by agents navigating in an olfactory environment.

Reviewer #2:

- Firstly, assumptions of Gaussian profiles in Assumptions 1 and 4 could be justified when it is first mentioned by referring to Figures 5 and 6. And those plots could be further analyzed to explicitly check the Gaussian nature, by standardizing and then plotting on a log scale to see if the log density falls quadratically.

Our assumption of Gaussianity was made primarily for mathematical tractability, and we did not expect it to necessarily hold in the plume due to the known non-Gaussian nature of turbulence. Nevertheless, we appreciate the need to test our assumptions, so we have now provided tests of each of our five assumptions in the supplementary material. We thank the reviewer for suggesting an approach for testing Gaussianity. That approach works best for univariate data (like the marginals of the data in the Figures the reviewer indicates). However, testing our assumptions required us to deal with bivariate data (when analyzing coefficients from a single source) and 4-variate data (when analyzing coefficients from pairs of sources), so instead we used energy statistics to compare distributions. We found that all of our assumptions except for the assumption of Gaussianity, broadly held at the nine probe locations we tested in the plume generated sources arranged cross-stream. We hope these tests address the reviewer's inquiry about the validity of our assumptions.

- Assumption 2 in particular, and how spatial dependence in general is handled in this work, is presented in a rather confusing manner. It seems that the ideas and words “source”, “source separation” and “location” are used somewhat interchangeably, when they should be treated separately.

We are very grateful to the reviewer for catching this oversight. Upon examining that section of the manuscript we found that we indeed had confused source location and source separation in some of our descriptions. We have therefore partially rewritten the section stating our assumptions, reordered them to clarify the presentation and have included detailed derivations linking source locations to intersource distance in Methods Section 5.3.3. Through this clarification process we realized that we needed another assumption, that the conditional distribution of coefficients from one source given those at another depends only on the intersource distance. We hope that our revised statement of our assumptions and their implications clarifies our previous presentation.

- In general, if these results are expected to hold in different locations of the plume relative to the source locations then this should explored and justified more, eg. by analyzing the distribution of the Fourier coefficients or r_n at different locations in the plume...It may be that things become much more complicated off-center, but at least if a few samples are taken at different, significantly spaced, downwind locations along the centerline and similarly analyzed, this will make the work more general.

We thank the reviewer for this crucial point about the generalizability of our results. This question was also the first point raised by Reviewer 1, and we refer the reviewer to our answer there.

- Additionally, it is not clear to me how the assumption of the form of rho_n in equation 18 is linked to the assumption in equation 33.

We apologize to the reviewer for the confusion. Equation 18 (now Equation 15) defines rho_n for the given intersource distances, as the in-phase correlation for all sources that distance apart. In principle, that value can be arbitrarily different at different intersource distances - equation 18 is not meant to constrain the dependence on intersource distance in any way, only to define the correlation at a given intersource distance. Subsequent derivations reveal that Fisher information is determined by how rho_n changes with intersource distance, and we chose the form in Equation 33 (now Equation 24) purely phenomenologically. We have added text immediately following Equation 15 to make this clarification.

- Justification of exponential dependence, eg. in Fig 8 could also be more carefully demonstrated by plotting in log-scale

We thank the reviewer for the suggestion of assessing the exponential fits on a log-scale. If we understand their suggestion correctly, exponential decay fits with offset, of the form in Equation 33 (now Equation 24) can be assessed by plotting the logarithm of the ratio of offset-subtracted correlations to 1 minus the offset, against the independent variable, in this case the intersource distance. A perfect fit would reveal a linear trend with slope -1/gamma, and this could be compared to the data being fit. Individual bootstrap fits could certainly be assessed in this way. In the panels of Fig 8A-D we are overlaying the results of multiple bootstrap fits (gray traces), but this could be done along these lines by e.g. plotting the data from each fit in a different colour, at the risk of a messier plot. However, the main problem is how to also overlay the bootstrap medians and confidence intervals of the data (the coloured traces in Figure 8A-D) on the plots assessing the fits. Doing so would require subtracting an offset from each data point (e.g. the median at a given intersource distance), before computing ratios, taking logarithms etc. - but which offset should we use? Perhaps the reviewer can clarify this for us.

Minor comments:

- Line 14, “correlation of odor concentration fields” is not very clear. Maybe “correlation of odor concentration timeseries."

Thank you, done.

- Typos in lines 88 and line 196, and line 235

Thank you, corrected.

- Instead of denoting as information between n and s, might be better to denote as information between r_n and s, but I’m happy to let the authors decide

Thank you! The reviewer's recommendation is indeed clearer and we have changed our notation accordingly.

- The purpose of the analysis of the surrogate data could be made more clear. Maybe a little more in the main text about how it is generated will shed more light on generally what makes the spectral distribution of information flat vs. non-flat.

We apologize for the lack of clarity about the surrogate datasets - our description in the Main Text is indeed too terse. We have now expanded Section 2.3.1 to better elucidate how the surrogate datasets are generated and used as ground-truth for different distributions of information.

- The motivation behind the study and ethological relevance of the findings could still be made stronger. Perhaps further expansion in the introduction of the findings in citation [35] and how resolving source separations at such a scale can matter in real contexts around line 361.

We have now rewritten our introduction to better motivate our work in the context of olfactory search. We hope that this helps clarify the relevance of our findings to real world contexts.
